# Identifying hospital-level predictors for antibiotic use: a Global Point Prevalence Survey study among Belgian, Philippine and South African hospitals

**DOI:** 10.1093/jacamr/dlag042

**Published:** 2026-04-03

**Authors:** Annelies Boven, Anna Ivanova, Ann Versporten, Ines Pauwels, Rhenalyn Bo, Jemelyn Garcia, Mari Rose De Los Reyes, Reshma Misra, Heather Finlayson, Shaheen Mehtar, Nele Brusselaers, Erika Vlieghe, Global-PPS participants, Agnes Yabut, Aleder Therese Dumapay, Andries Gous, Anne-Marie Van Den Abeele, Anthony Ceraos, Antoinette Moolman, Ashendri Pillay, Ashmika Gangadin, Baptist Declerck, Bart Glibert, Bashira Adam, Bea Van den Poel, Bernadett Gosnell, Beverley Hooper, Camille Enriquez, Carine Jaumotte, Carmina Joy Laggui, Catherine Torres-Jison, Cecilia Govindasamy, Christelle Meuris, David Paul Moore, Elizabeth Hoffman, Eugenne Elliott, Eva Rutten, Evan Eriele Dy, Fhebie Zapanta, Graeme Hofmeyr, Graziella May Revilla, Gugu Goge, Helena Smit, Israel Ramokgadi, Jacques D Du Toit, Jemelyn Garcia, Jeremy Nel, Jhonric Landoy, John Andrew Camposano, Jonh Paul Javier, Joy Andres, Justine Emma Rose Vergara-Silvano, Kai Daryl Bocala, Karen Loise Tarnate, Kristof Bafort, Lebogang Brenda Molepo, Liezel Afaga, Louis Ide, Lozel Villadore, Ma Theresa Diwata Gagan, Madnieyah Salasa, Maria Katrina Rayos, Mariss Catherine Roque, Mary Crist Jamora, Mihloti Pister Mathonsi, Nectarios Papavarnavas, Nirupa Misra, Nomi Aparece, Nomusa Mkhabela, Nondumiso Sibisi, Ntombifuthi Bophela, Olwethu Ncume, Paul-Emile Claus, Sandrine Jacquet, Sanele Mngomezulu, Sarah Resseler, Sharon Bucoy, Shayne Julieane Morales, Shingie Matema, Stephen Rae Fontanilla, Stijn Jonckheere, Tasneem Waja (Mukadam), Tharushni Perumal, Theresa Yoro, Vanessa Aira Rivera, Veerle Westelinck, Vita Gasataya, Welcome Sihle Mbatha, Xavier Holemans, Ysaline Seynaeve, Zesca Meyer, Zingisa Mandlana, Ziningi Ntuli

**Affiliations:** Global Health Institute, University of Antwerp, Antwerp, Belgium; Interuniversity Institute for Biostatistics and Statistical Bioinformatics (I-BioStat), Data Science Institute, Hasselt University, Hasselt, Belgium; Global Health Institute, University of Antwerp, Antwerp, Belgium; Global Health Institute, University of Antwerp, Antwerp, Belgium; Research Institute for Tropical Medicine, Filinvest Corporate City, Alabang, Muntinlupa, Philippines; Research Institute for Tropical Medicine, Filinvest Corporate City, Alabang, Muntinlupa, Philippines; Research Institute for Tropical Medicine, Filinvest Corporate City, Alabang, Muntinlupa, Philippines; KwaZulu-Natal Department of Health, Durban, South Africa; Department of Paediatrics and Child Health, Stellenbosch University, Stellenbosch, South Africa; Infection Control Africa Network, Cape Town, South Africa; Infection Control Technical Working Group of the Ministerial Advisory Committee on AMR, Cape Town, South Africa; Unit for Infection Prevention and Control (UIPC), Faculty of Health Sciences, Stellenbosch University, Cape Town, South Africa; Global Health Institute, University of Antwerp, Antwerp, Belgium; Department of Women’s and Children’s Health, Karolinska Institutet, Stockholm, Sweden; Global Health Institute, University of Antwerp, Antwerp, Belgium; Department of General Internal Medicine, Infectious Diseases and Tropical Medicine, University Hospital Antwerp, Antwerp, Belgium

## Abstract

**Introduction:**

Understanding determinants that influence antibiotic use is crucial for developing effective stewardship interventions that optimize the use of antibiotics and ultimately mitigate the burden of antibiotic resistance. This study aims to find hospital-level predictors of antibiotic use among hospitals worldwide.

**Methods:**

Antibiotic use data were collected from hospitals that participated in 2022–23 in the Global Point Prevalence Survey (Global-PPS) basic inpatient and Healthcare-Associated Infection (HAI) modules from three countries with high participation degrees: Belgium, the Philippines and South Africa. A linear mixed model was applied to predict hospital-level prevalence, considering all variables from the Global-PPS as potential predictors. Stepwise forward and backward selection modelling were conducted to identify the best-fitting models per country separately.

**Results:**

In total, data from 138 hospitals were retrieved, including 19 Belgian, 55 Philippine and 64 South African hospitals. No predictors were shared across the three countries, such as hospital type, proportion of occupied medical beds and proportion of available guidelines. However, the proportions of admitted patients with certain invasive devices were significant predictors for increased odds of hospital-wide antibiotic prescribing, despite the type of invasive device varying between countries.

**Conclusions:**

Factors related to the patient mix, including the proportion of patients with an invasive device, are associated with increased odds of antibiotic prescribing at hospital level. Considering substantial differences in predictors associated with country-specific patient populations and hospital characteristics, more research is needed to explore additional determinants, such as healthcare system and broader contextual factors that may influence hospital-level antibiotic prevalence.

## Introduction

Antibiotic resistance is a globally recognized major healthcare threat, with over one million associated deaths in 2021 only,^1^ and is mostly attributed to the misuse and overuse of antibiotics worldwide. Understanding the factors that drive antibiotic use is crucial for developing effective interventions that might optimize the use of antibiotics and ultimately mitigate the burden of antibiotic resistance.

Previously identified factors associated with increased antibiotic use among admitted patients include several patient characteristics, such as male sex,^2–5^ racial groups,^5^ age group^6^ and religion,^6^ as well as age group of the prescriber.^7^ Identified factors related to the healthcare system and setting include the availability of clinical guidelines,^6^ several financial and economic factors,^6,8^ health and transport infrastructure,^6^ and relative humidity.^6^

Notably, there is a lack of studies identifying organization-level predictors of antibiotic use, such as hospital characteristics like patient mix. Such factors can also be useful for hospitals to estimate and compare their antibiotic prescribing patterns with those of other institutions. However, large heterogeneity in antibiotic prescribing patterns,^9^ and the wide variety in data collection techniques, may influence these organization-level predictors of antibiotic use. Therefore, this study aims to find predictors for antibiotic prescribing among hospitals of three different countries: Belgium, the Philippines and South Africa, taking their heterogeneity into consideration.

## Methods

### Included data

Cross-sectional antibiotic prescribing data were gathered from the Global Point Prevalence Survey (Global-PPS) inpatient dataset. We selected hospitals that had participated in the basic inpatient with the optional Healthcare-Associated Infection (HAI) module, which collects more detailed information on the use of invasive devices for all admitted inpatients and risk factors for antibiotic prescribing for those on at least one antibiotic. Furthermore, we restricted inclusion to hospitals from countries where at least 10 hospitals had participated in the years under review to maximize statistical power. Consequently, we retained 19 hospitals from Belgium, 55 from the Philippines (both year 2022), and 64 from South Africa (year 2023) (Table 1). The inpatient protocol and data collection templates are described in Appendices I and II.

**Table 1.** Hospital and patient characteristics used in the linear mixed model to identify important predictors for antibiotic use in Belgian, Philippine and South African hospitals

Characteristics		Belgium		Philippines		South Africa
*N* hospitals		19		55		64
*N* surveys (Basic + HAI module)^a^		19		100		68
Years		2022		2022		2023
*N* admitted patients		4722		20 131		10 875
*N* antibiotic prescriptions		1603		15 074		4816
*N* patients treated with antibiotics		1407		10 384		3482
Antibiotic prevalence		29.8%		51.6%		32.0%
*N* adults treated with antibiotics		1323		7333		2277
*N* children treated with antibiotics		80		1971		819
*N* neonates treated with antibiotics		4		1080		386
Mean age of treated patients in years (SD)		66.1 (22.6)		36.1 (26.5)		31.0 (25.1)
Median age of treated patients in years (IQR)		72 (58–83)		35 (12–58)		31 (3–50)

Top 5 most common comorbidities among patients treated with antibiotics (*n*, %)	Diabetes mellitus	261 (19.7)	Diabetes mellitus	1232 (16.8)	AIDS/HIV	445 (19.5)
	Haematological or solid cancer	230 (17.4)	Chronic Renal Disease	693 (9.5)	Diabetes mellitus	277 (12.2)
	Chronic lung disease	217 (16.4)	Haematological or solid cancer	598 (8.2)	Haematological or solid cancer	153 (6.7)
	Chronic neurological conditions	206 (15.6)	Chronic lung diseases	447 (6.1)	Tuberculosis	124 (5.4)
	Chronic renal disease	164 (12.4)	Tuberculosis	277 (3.8)	Chronic renal disease	105 (4.6)

**Candidate predictors**						
*N* teaching hospitals (%)			5 (26.3)	31 (56.4)		30 (46.9)
*N* primary or secondary (‘general’) hospitals (%)			18 (94.7)	11 (20.0)		44 (68.8)
*N* tertiary hospitals (%)			1 (5.3)	40 (72.7)		18 (28.1)
*N* infectious diseases hospital (%)			0 (0.0)	2 (3.6)		0 (0.0)
*N* other hospitals (%)			0 (0.0)	2 (3.6)		2 (3.1)
*N* hospitals with ICU, transplant or ID ward (%)			17 (89.5)	53 (96.4)		39 (60.9)
*N* hospitals with obstetrics or gynaecology ward (%)			10 (52.6)	24 (43.6)		34 (53.1)
*N* hospitals with haemato-oncology ward (%)			7 (36.8)	13 (23.6)		7 (10.9)
*N* hospitals with long-term care, geriatric, or rehabilitation wards (%)			15 (78.9)	1 (1.8)		0 (0.0)
*N* beds (median, IQR)			336 (196–402.5)	424 (223.5–729)		147.5 (76.75–261.75)
*N* surgical beds (median, IQR)			77 (52.5–92)	113 (37–208.5)		34.5 (10.5–90.5)
*N* medical beds (median, IQR)			206 (138.5–300)	281 (137.5–437)		84 (49.25–167.25)
*N* ICU beds (median, IQR)			12 (10.5–18.5)	51 (24–104)		5.5 (0.0–28.0)
*N* occupied beds (median, IQR)			66 (52–89.5)	140 (66.5–276.0)		35.5 (20.25–69.25)
*N* occupied surgical beds (median, IQR)			0 (0–2.5)	0 (0–8)		0 (0–0)
*N* occupied medical beds (median, IQR)			45 (29.5–59)	101 (53–180)		25 (10.75–40.5)
*N* occupied ICU beds (median, IQR)			4 (2–6.5)	18 (9–37.5)		3 (0–13)
*N* admitted adult patients (%)			4404 (93.3)	15 256 (75.8)		8285 (76.2)
*N* admitted paediatric patients (%)			251 (5.3)	3207 (15.9)		1540
*N* admitted neonatal patients (%)			67 (1.4)	1668 (8.3)		1050

***Patients with… among all admitted patients***						
Urinary catheter		*N* patients (%)	683 (14.5)		2888 (14.3)	1646 (15.1)
		Median (IQR)	7 (1–15)		4 (0–15)	3 (0–9)
Peripheral vascular catheter		*N* patients (%)	2149 (45.5)		14 302 (71.0)	4592 (42.2)
		Median (IQR)	18 (5.5–34)		29 (9–73)	13 (5–29)
Central vascular catheter		*N* patients (%)	367 (7.8)		1377 (6.8)	539 (5.0)
		Median (IQR)	4 (1–7.5)		1 (0–4)	0 (0–3)
Non-invasive mechanical ventilation		*N* patients (%)	44 (0.9)		935 (4.6)	274 (2.5)
		Median (IQR)	0 (0–1)		1 (0–4)	0 (0–1)
Invasive respiratory endotracheal intubation		*N* patients (%)	60 (1.2)		1059 (5.3)	319 (2.9)
		Median (IQR)	0 (0–1)		1 (0–5)	0 (0–1)
Inserted tubes and drains		*N* patients (%)	348 (7.4)		1921 (9.5)	556 (5.1)
		Median (IQR)	2 (0.5–7)		3 (0–9)	0 (0–2)

***Number of patients with… among all patients receiving antibiotics (%)***						
Treatment based on biomarker^b^		137 (58.1)		—		—
Targeted antibiotic treatment		464 (33.0)		1269 (12.2)		446 (12.8)
Community-acquired infections		834 (59.3)		4949 (47.7)		1865 (53.6)
Healthcare-associated infections		315 (22.3)		1893 (18.2)		768 (22.1)
Previous ICU hospitalization		25 (1.7)		211 (2.0)		174 (5.0)
Previous non-ICU hospitalization		402 (28.6)		1418 (13.7)		798 (22.9)
Ultimately/rapidly fatal disease (McCabe score)^10^		267 (19.0)		1806 (17.4)		537 (15.4)
Surgical procedures during current admission		489 (34.7)		2901 (27.9)		947 (27.2)
Previous antibiotic use		364 (25.9)		1430 (13.8)		757 (21.7)
Male patients		707 (50.3)		5097 (49.1)		1697 (48.7)
Multiple comorbidities		327 (23.2)		741 (7.1)		266 (7.6)
***Number of antibiotic prescriptions with… (%)***						
Existing guidelines among antibiotic prescriptions^c^		1566 (94.4)		14 505 (96.2)		4430 (92.0)
Documented reason for prescription^c^		1405 (89.7)		11 841 (78.6)		3977 (82.6)
Recorded stop/review date^c^		863 (55.1)		8233 (54.6)		1779 (36.9)

^a^Hospitals participated in maximum 3 surveys per year.

^b^Biomarker data are excluded from analyses for the Philippines and South Africa due to the amount of missing data.

^c^Unknown values are excluded from the analyses.

### Model development

A linear mixed model^11^ was applied to predict hospital-level prevalence of antibiotics (ATC code J01) per country separately with hospital as a random effect to correct for the association of the measurements within hospitals. The logit-transformed prevalence was chosen as primary outcome of the model to limit the prevalence between 0 and 1, since it was defined as a proportion. An alternative way of modelling, such as logistic regression, was not possible as detailed information of the patients without antimicrobial use was not included in the Global-PPS protocol.

All available variables collected in the Global-PPS dataset were considered as candidate predictors, apart from treatment regimen and prophylaxis type (medical or surgical) (Appendix III). We retained the proportion of patients receiving antibiotics with a community-acquired infection (CAI) or HAI as predictors, even though they were derived from the same underlying variable as the prophylaxis types, because both factors are not necessarily direct characteristics of the treatment but might represent important characteristics of the patient mix and hence influence overall antibiotic prescribing at hospital-level.

All predictors were calculated at hospital- or ward-level (Appendix III). Hospital-level variables included level of care (‘hospital type’), teaching status, presence of certain ward types, total number of surveyed beds and bed occupancy rate, while ward-level variables include detailed information on the admitted patients, such as proportion of patients receiving targeted antibiotic treatment, patients with at least one invasive device, patients with multiple morbidities, etc. (Table 1 and Appendix III).

Candidate predictors were included in the first step of the analysis if the proportion/prevalence was non-missing. Predictors were excluded if there was more than 50% missingness, or if the predictor was highly correlated with another variable, defined as having a Pearson ’s or Spearman’s correlation of 0.95 or higher. From the two highly correlated predictors, the easiest interpretable one was selected.

A final model was built for each country separately by forward model selection and backward extraction. In forward model selection, starting with a univariate linear mixed model, predictors were added one by one to the model, beginning with the predictor that had the lowest *P* value. Predictors were added to the forward model using likelihood ratio tests with a significance level of 20%.

In backward extraction, the predictors were excluded one by one from the model obtained from forward selection, starting with the least influential predictor, until all remaining model predictors were significant at level 5%. The likelihood ratio test was applied to determine significance.

To facilitate interpretation of the regression coefficients, we exponentiated the coefficients of the final model, which included all selected predictors, to compute odds ratios (ORs). These represent the relative change in the odds of hospital-wide antibiotic utilization associated with a one-unit increase in each predictor, conditioned on the other predictors. For predictors expressed as proportions, we additionally computed ORs for 10% increments, calculated as OR^0.1. These increments were chosen to facilitate clinical interpretation, since 100% increments for proportional predictors are clinically improbable and generate extreme, difficult-to-interpret estimates. All analyses were carried out in R (version 4.3.1).

## Results

In total, 138 hospitals participating in 2022 and 2023 were included, of which 19 Belgian hospitals, 55 Philippine hospitals and 64 South African hospitals (Table 1). Data were merged at hospital level for hospitals that had participated multiple times within one year. One (0.7%) hospital declined participation in this study.

### Belgian model

Most Belgian hospitals included in this study were general hospitals (94.7%). In total, 4722 admitted patients and 1407 patients treated with an antibiotic were surveyed (Table 1). Most patients were admitted to adult wards (93.3%) (Table 1). Peripheral vascular catheters were inserted in 45.5% of the admitted patients, and urinary catheters in 14.5% (Table 1). Among patients treated with an antibiotic, 50.3% were male, 34.7% underwent a surgical procedure during their current admission, 22.3% had an HAI, and 19.0% had ultimately or rapidly fatal disease according to the McCabe score (Table 1). For most antibiotic prescriptions, guidelines were available (94.4%), a reason for the treatment was documented (89.7%) and a stop/review date was recorded (55.1%) (Table 1).

**Figure 1.**
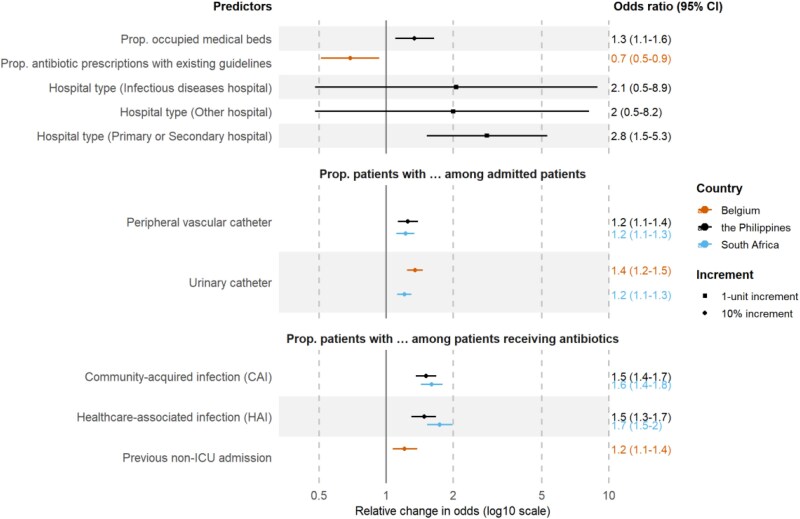
Relative change in the odds of hospital-wide antibiotic utilization associated with a one-unit and 10% increase in each predictor from a linear mixed model among 138 Belgian, Philippine and South African hospitals. Values are presented on a logarithmic scale (base 10). Abbreviations: Prop. = proportion.

Our final model including 19 Belgian hospitals indicated that a 10% increase in the proportion of admitted patients with a urinary catheter was associated with a 35% increase in the odds of hospital-wide antibiotic utilization (OR = 1.35, 95% CI: 1.24–1.46), compared to a 21% increase in these odds per 10% increase in the proportion of treated patients with a previous non-ICU admission (OR = 1.21, 95% CI: 1.07–1.38), and a 31% decrease in the odds per 10% increase in the proportion of antibiotic prescriptions with existing guidelines (OR = 0.69, 95% CI: 0.51–0.93) (Table 2 and Figure 1).

**Table 2.** Relative change in the odds of hospital-wide antibiotic utilization associated with a one-unit and 10% increase in each predictor from a linear mixed model among 19 Belgian hospitals

Predictor	*N* (%)	Coefficient (95% CI)	Relative change in odds (95% CI)	Relative change in odds for 10% increments (95% CI)	*P* value^a^
Proportion of antibiotic prescriptions with existing guidelines	1566 (94.4)	−3.72 (−6.72 to 0.69)	0.02 (0.00–0.50)	0.69 (0.51–0.93)	0.020
Proportion of patients with a previous hospital (non-ICU) admission among patients receiving antibiotics	402 (28.6)	1.94 (0.66–3.21)	6.94 (1.94–24.84)	1.21 (1.07–1.38)	0.003
Proportion of admitted patients with a urinary catheter	683 (14.5)	2.97 (2.14–3.81)	19.47 (8.47–45.27)	1.35 (1.24–1.46)	0.000

^a^Applies to both relative changes in odds.

### Philippine model

Most Philippine hospitals were tertiary hospitals (72.7%) (Table 1). A total of 20 131 admitted patients and 10 384 patients treated with an antibiotic were surveyed. A majority of patients were admitted to adult wards (75.8%) (Table 1). Peripheral vascular catheters (71.0%) and urinary catheters (14.3%) were frequently inserted in admitted patients. Among patients treated with an antibiotic, 49.1% were male, 27.9% underwent a surgical procedure during their current admission, 17.4% had an ultimately or rapidly fatal disease (McCabe score), 13.8% had previously received antibiotics, and 18.2% had an HAI (Table 1). For many antibiotic prescriptions, guidelines were available (96.2%), a reason for the treatment was recorded (78.6%), and a stop/review date was documented (54.6%) (Table 1).

In the final model containing data from 55 Philippine hospitals, a 10% increase in the proportion of patients with a CAI among those receiving antibiotics was associated with a 51% increase in the odds of hospital-wide antibiotic utilization (OR = 1.51, 95% CI: 1.36–1.68) (Table 3 and Figure 1). This increase in odds was 48% per 10% increase in the proportion of patients with a HAI (OR = 1.48, 95% CI: 1.30–1.67), 34% for the proportion of occupied medical beds (OR = 1.34, 95% CI: 1.10–1.64), and 25% for the proportion of admitted patients with a peripheral vascular catheter (OR = 1.25, 95% CI: 1.13–1.39) (Table 3). Compared to tertiary care hospitals, primary or secondary hospital types was associated with a 2.8-fold increase in the odds of hospital-wide antibiotic utilization (OR = 2.83, 95% CI: 1.52–5.29), while infectious disease and other hospital types showed no significant change in these odds (Table 3).

**Table 3.** Relative change in the odds of hospital-wide antibiotic utilization associated with a one-unit and 10% increase in each predictor from a linear mixed model among 55 Philippine hospitals

Predictor	*N* (%)	Coefficient (95% CI)	Relative change in odds (95% CI)	Relative change in odds for 10% increments (95% CI)	*P* value^a^
Hospital type (Primary or Secondary hospital)	11 (20.0)	1.04 (0.42–1.67)	2.83 (1.52–5.29)	—	0.002
Hospital type (Infectious diseases hospital)	2 (3.6)	0.72 (−0.74–2.18)	2.06 (0.48–8.87)	—	**0**.**330**
Hospital type (Other hospital)	2 (3.6)	0.69 (−0.73–2.10)	2.00 (0.48–8.15)	—	**0**.**331**
Proportion occupied medical beds	45 (29.5–59)	2.95 (1.00–4.97)	19.20 (2.71–142.72)	1.34 (1.10–1.64)	0.004
**Proportion of patients with … among patients receiving antibiotics**					
Community-acquired infections as indication	4949 (47.7%)	4.14 (3.11–5.17)	62.78 (22.38–175.67)	1.51 (1.36–1.68)	0.000
Healthcare-associated infections as indication	1893 (18.2%)	3.91 (2.66–5.14)	49.87 (14.30–171.20)	1.48 (1.30–1.67)	0.000
**Proportion of admitted patients with … among all admitted patients**					
Peripheral vascular catheter	14 302 (71.0%)	2.25 (1.20–3.29)	9.49 (3.33–26.89)	1.25 (1.13–1.39)	0.000

^a^Applies to both relative changes in odds.

### South African model

A total of 10 875 admitted patients and 4816 patients treated with an antibiotic were surveyed, mostly from general hospitals (68.8%) (Table 1). Many admitted patients stayed in adult wards (76.2%) and had a peripheral vascular catheter (42.2%) and a urinary catheter (15.1%). Among patients treated with an antibiotic, 48.7% were male, 27.2% underwent a surgical procedure during current admission, 21.7% previously received antibiotics, 22.1% had an HAI, and 15.4% had an ultimately or rapidly fatal disease according to the McCabe score (Table 1). Guidelines were available for 92.0% of all antibiotic prescriptions, a reason for the treatment was recorded for 82.6%, and the stop/review date was recorded for 36.9% (Table 1).

In the final model for South Africa, including 64 hospitals, a 10% increase in the proportion of patients with an HAI among those receiving antibiotics was associated with a 74% increase in the odds of hospital-wide antibiotic utilization (OR = 1.74, 95% CI: 1.53–1.98) (Table 4 and Figure 1). This increase in odds was 60% per 10% increase in the proportion of patients treated for a CAI (OR = 1.60, 95% CI: 1.43–1.79), 22% for the proportion of admitted patients with a peripheral vascular catheter (OR = 1.22, 95% CI: 1.11–1.34), and 21% for the proportion of admitted patients with a urinary catheter (OR = 1.21, 95% CI: 1.12–1.34) (Table 4).

**Table 4.** Relative change in the odds of hospital-wide antibiotic utilization associated with a one-unit and 10% increase in each predictor from a linear mixed model among 64 South African hospitals

Predictor	*N (%)*	Coefficient (95% CI)	Relative change in odds (95% CI)	Relative change in odds for 10% increments (95% CI)	*P* value^a^
**Proportion of patients with … among patients receiving antibiotics**					
Community-acquired infections as indication	1865 (53.6)	4.70 (3.60–5.81)	110.3 (36.53–333.03)	1.60 (1.43–1.79)	0.000
Healthcare-associated infections as indication	768 (22.1)	5.54 (4.25–6.83)	225.21 (69.97–929.09)	1.74 (1.53–1.98)	0.000
**Proportion of patients with … among admitted patients**					
Urinary catheter	1646 (15.1)	1.88 (1.16–2.61)	6.57 (3.18–13.58)	1.21 (1.12–1.30)	0.000
Peripheral vascular catheter	4592 (42.2%)	1.97 (1.00–2.94)	7.17 (2.73–18.88)	1.22 (1.11–1.34)	0.000

^a^Applies to both relative changes in odds.

The estimates of the linear mixed model and the type III ANOVA tests for all three countries are included in the supplementary materials (Appendices IV and V) (available as Supplementary data at *JAC-AMR* Online).

## Discussion

While many point prevalence studies have described local, national, or cross-country antibiotic prescribing patterns, few have explored the underlying determinants that drive these practices, particularly across multiple countries. This study, therefore, investigated predictors for hospital-wide antibiotic utilization among 138 hospitals in Belgium, the Philippines and South Africa.

From the Global-PPS dataset, we identified no common predictors associated with increased odds of hospital-wide antibiotic utilization across all three countries, although higher proportions of admitted patients with invasive devices seemed key factors. These and other factors related to the patient mix were associated with increased odds of hospital-wide antibiotic utilization in this study: in Belgium, key factors include higher proportions of admitted patients with a urinary catheter and patients with a previous non-ICU admission among those who received antibiotics. In both the Philippines and South Africa, identified predictors include higher proportions of patients treated for a CAI or HAI, as well as increased proportions of admitted patients with a peripheral vascular catheter. In South Africa, elevated proportions of admitted patients with urinary catheters were additionally associated with greater odds of hospital-wide antibiotic utilization.

These findings are not surprising, as antibiotics are frequently prescribed in patients with invasive devices,^12,13^ CAIs^14^ or HAIs.^15,16^ These predictors might reflect a greater case complexity in wards with higher antibiotic prevalences, which is consistent with previous research that found a correlation between in-hospital antibiotic use and chronic use of urinary catheters,^2^ and between in-hospital antibiotic use and case mix index.^17,18^ Nevertheless, invasive devices like peripheral vascular catheters are commonly used to administer (parenteral) antibiotics,^19,20^ or may be used concurrently with antibiotic treatments to manage ongoing infections, which may alternatively explain the increased odds of antibiotic use. Notwithstanding, we advise caution when interpreting the association between increased odds of hospital-wide antibiotic use and elevated proportions of patients receiving antibiotics for a CAI or HAI, as this might reflect an association between antibiotic prevalence and therapeutic prescribing rather than with characteristics of the patient mix.

Other factors related to the patient mix of a hospital were not associated with antibiotic use, though this could be attributed to the smaller number of specific cases included in the model, such as the proportion of admitted patients with a central vascular catheter, an invasive respiratory endotracheal intubation, tubes and drains and non-invasive mechanical ventilation. While the proportion of patients receiving antibiotics with ultimately or rapidly fatal disease (according to the McCabe score), multiple comorbidities, a surgical procedure during current admission, or sex were not identified as predictors for hospital-level antibiotic use in these models, previous studies have reported increases in antibiotic use associated with chronic use of central venous catheters,^2^ prior antibiotic use,^3,4^ previous hospitalization,^3^ comorbidities,^3,4^ and male sex.^2–5^ One explanation is the difference in setting and study design, as these studies were conducted solely in high-income countries using logistic or negative binomial mixed regression applied on longitudinal cohort data.^2–5^ However, considering we use cross-sectional data, it is possible that we lacked sufficient power to identify all important predictors for hospital-wide antibiotic utilization.

This study additionally identified several hospital-level predictors. In Belgium, a higher proportion of existing guidelines among all antibiotic prescriptions was associated with decreased odds of hospital antibiotic utilization. The Infectiology Guide of the Belgian Society for Infectiology and Clinical Microbiology is nationally widely adopted,^21^ and also serves as a reference for the harmonization of local antibiotic guidelines which were supported financially through the HOST projects conducted between 2021 and 2025.^22,23^ In line with previous research that has established the importance of antibiotic guideline implementation,^24^ this finding suggests that the availability of guidelines curbs (unnecessary) antibiotic use in hospitals and highlights the need for freely available national guidelines for in-hospital antibiotic use in Belgium.

In the Philippines, elevated proportions of occupied medical beds were associated with significant increases in the odds of hospital antibiotic utilization. A study investigating 16 Swiss long-term care facilities described a similar finding: they reported a positive association between the number of occupied beds and antibiotic use.^25^ This might suggest that higher occupancy rates, which potentially reflect higher patient turnover, influence antibiotic use. This effect might be direct, for instance when antibiotics are prescribed to treat HAIs transmitted in crowded wards,^26^ or indirect, through increased time pressure on healthcare staff.^27^ The influence of overcrowding in hospitals on the decrease in infection, prevention and control (IPC) and antimicrobial stewardship (AMS) measures has additionally been observed during the COVID-19 pandemic.^28^

Additionally, primary or secondary hospital status was associated with increased odds of antibiotic utilization compared to tertiary hospitals in the Philippine model. Evidence from the literature is mixed: while some studies report higher antimicrobial use patterns among primary care hospitals and decreased antibiotic use among hospitals with a university affiliation,^12,29^ other studies found no notable differences between hospital types.^30,31^

Interestingly, apart from hospital type, the availability of clinical guidelines, and number and occupancy rate of medical beds, other ward- and hospital-level factors were not associated with significant changes in odds of antibiotic utilization, such as the teaching status and the presence of haemato-oncology wards and of long-term care, geriatric or rehabilitation wards. Limited power may have obscured some key determinants of hospital-level antibiotic use.

These findings have several implications. First, the association between hospital-wide antibiotic use and elevated proportions of patients with HAIs or invasive devices underscores the importance of IPC measures, to reduce the transmission or incidence of HAIs and ultimately curb antibiotic use. Second, the considerable heterogeneity in the predictors for antibiotic prescribing between the three countries suggests that, with the current data, a uniform model cannot be developed. There may be a need to broaden certain predictors to reduce the variability between countries and enhance robustness, e.g. by combining all invasive devices into one variable. Moreover, other factors may play a role in the hospital’s antibiotic utilization. A recent systematic review identified gender, education level and health status of patients, as well as the work experience of prescribing physicians, as significant factors for hospital antibiotic prescribing.^32^ Furthermore, it seems that AMS programmes are additionally associated with reduced antibiotic use in inpatient and outpatient care.^33^ This suggests hospital-level structure and process indicators on AMS are important in surveillance activities.

Although future research could consider more variables in the prediction modelling, for instance by incorporating variables from additional healthcare-related or socio-economic databases into the model, we believe that integrating prediction modelling in feedback from surveillance studies on antibiotic use deserves greater emphasis, to optimize comparison with similar hospitals and ultimately facilitate integration of antibiotic stewardship recommendations. Therefore, future work should involve refining and integrating prediction models in antibiotic surveillance feedback reports.

An important strength of this study was exploring the potential of point prevalence use data for identifying hospital-level predictors for antibiotic use to ultimately enhance comparability of point prevalence data between hospitals from different settings. We used a stepwise method for variable selection to consider the association between predictors and prevent loss of information.

Other strengths of this study were the use of a large database containing detailed data on antibiotic prescribing worldwide. Furthermore, the standardized method for data collection allowed for comparison between heterogeneous settings.

Limitations to this study include the lack of detailed patient data for patients who did not receive an antimicrobial prescription. While this is inherent to the Global-PPS design, it impairs the prediction of antibiotic prescribing at patient-level in this study and potentially biases the prevalence of patients with certain comorbidities or indications. This limitation was mitigated by computing the prediction at ward- and hospital-level, which provides insightful estimates for stewardship efforts, and by limiting the prevalence of patients with certain comorbidities or indications to the patients who received antibiotic treatment. Moreover, we lack accurate information on the severity of infections, which is likely an important predictor for hospital-wide antibiotic use.

Next, the results may not be fully generalizable to the inpatient care settings in Belgium, the Philippines and South Africa, due to the use of cross-sectional data across hospitals and networks that participated on voluntary basis in Belgium (19.4% of the total 103 hospitals in 2022),^34^ the Philippines (approximately 4.6% of at least 1200 licensed hospitals in 2022),^35^ and South Africa (11.7% of the total 544 hospitals in 2023).^36^ This could result in non-response bias. Furthermore, clustering at hospital- or ward-level could influence the results, although this effect was mitigated by modelling hospitals as a random effect. Clustering could be further influenced by measurement bias, although we tried to minimize this effect by using a simple protocol with automated data validation checks during data entry, and by providing regular online training webinars with the opportunity to ask any questions during or after the training.

In addition, a limitation of this study was the lack of information on important known predictors for antibiotic use, including country-wide burden of antibiotic resistance, health economic status, hospitals’ resources and capacities, seasonality, and broader contextual factors. The use of cross-sectional data rather than longitudinal data was an additional limitation. Including these broader contextual factors from detailed, longitudinal data, was out of the scope of this study: we aimed to find important predictors from a point prevalence study that hospitals could use to recognize whether they are at higher risk of antibiotic prescribing within their hospital or ward.

### Conclusion

In this study, we have explored the use of point prevalence data to identify important predictors for antibiotic prescribing practices. Among all potential predictors, only the proportion of invasive device utilization emerged as a shared key determinant of hospital-wide antibiotic prescribing across the three countries. Although the data were collected cross-sectionally and are not nationally representative for the three countries, the findings nonetheless offer valuable insights for future study. The observed variation in antibiotic prescribing predictors across the three countries underscores the need for deeper research to achieve more precise findings. Incorporating additional healthcare-related or socio-economic databases into the model could potentially yield more clinically relevant results at country level.

## Supplementary Material

dlag042_Supplementary_Data

## Members of Global-PPS participants

Agnes Yabut, Research Institute for Tropical Medicine, Muntinlupa City, Philippines. Aleder Therese Dumapay, University of Perpetual Help Medical Center Las Piñas, Las Piñas, Philippines. Andries Gous, Sefako Makgatho Health Sciences University, Ga-Rankuwa, South Africa. Anne-Marie Van Den Abeele, St-Lucas Hospital, Ghent, Belgium. Anthony Ceraos, Medical Center Muntinlupa Inc., Muntinlupa City, Philippines. Antoinette Moolman, National Health Laboratory Services Charlotte Maxeke Complex, Johannesburg, South Africa. Ashendri Pillay, Durban, KwaZulu-Natal, South Africa. Ashmika Gangadin, G J Crookes Hospital, Scottburgh, South Africa. Baptist Declerck, Ziekenhuis Oost-Limburg, Genk, Belgium. Bart Glibert, AZ Glorieux, Ronse, Belgium. Bashira Adam, Mthatha, South Africa. Bea Van den Poel, AZ Jan Portaels, Vilvoorde, Belgium. Bernadett Gosnell, Victoria Mxenge Hospital, Durban, South Africa. Beverley Hooper, Port Shepstone Regional Hospital, Port Shepstone, KwaZulu-Natal, South Africa. Camille Enriquez, Research Institute for Tropical Medicine, Muntinlupa City, Philippines. Carine Jaumotte, Helora, Hôpitaux de Mons/Constantinople et Warquignies, Mons, Belgium. Carmina Joy Laggui, Cagayan Valley Medical Center, Tuguegarao, Philippines. Catherine Torres-Jison, Corazon Locsin Montelibano Memorial Regional Hospital, Bacolod City, Philippines. Cecilia Govindasamy, KZN DOH-Mahatma Gandhi Memorial Hospital, Durban KwaZulu-Natal, South Africa. Christelle Meuris, CHRVerviers, Verviers, Belgium. David Paul Moore, School of Clinical Medicine, Faculty of Health Sciences, University of the Witwatersrand, Johannesburg, South Africa. Elizabeth Hoffman, Queen Nandi Regional Hospital, Empangeni, KwaZulu-Natal Province, South Africa. Eugenne Elliott, National Health Laboratory (NHLS), Kimberley, South Africa. Eva Rutten, AZ Voorkempen, Malle, Belgium. Evan Eriele Dy, Cardinal Santos Medical Center, San Juan, Metro Manila, Philippines. Fhebie Zapanta, Corazon Locsin Montelibano Memorial Regional Hospital, Bacolod City, Philippines. Graeme Hofmeyr, Port St Johns, EC, South Africa. Graziella May Revilla, Gov. Celestino Gallares Memorial Medical Center, Tagbilaran City, Philippines. Gugu Goge, Estcourt District Hospital, Estcourt, South Africa. Helena Smit, Newcastle Regional Hospital, Newcastle, South Africa. Israel Ramokgadi, Klerksdorp/Tshepong Tertiary Hospital, Klerksdorp, South Africa. Jacques D. Du Toit, Mrc/Wits Rural Public Health and Health Transitions Research Unit (Agincourt), School of Public Health, Faculty of Health Sciences, University of the Witwatersrand, South Africa, Acornhoek, South Africa. Jemelyn Garcia, Asian Hospital and Medical Center, Muntinlupa City, Philippines. Jeremy Nel, Helen Joseph Hospital, Johannesburg, South Africa. Jhonric Landoy, Manila Doctors Hospital, Manila City, Philippines. John Andrew Camposano, Western Visayas Medical Center, Iloilo City, Philippines. Jonh Paul Javier, Las Piñas, Philippines. Joy Andres, Bataan General Hospital and Medical Center, Balanga, Philippines. Justine Emma Rose Vergara-Silvano, Asian Hospital and Medical Center, Alabang, Muntinlupa, Philippines. Kai Daryl Bocala, Roxas Memorial Provincial Hospital, Roxas City, Capiz, Philippines. Karen Loise Tarnate, Our Lady of the Pillar Medical Center, City of Imus, Cavite, Philippines. Kristof Bafort, Noorderhartziekenhuis, Pelt, Belgium. Lebogang Brenda Molepo, Pietersburg Hospital, Polokwane, South Africa. Liezel Afaga, Ospital NG Muntinlupa, Muntinlupa City, Metro Manila, Philippines. Louis Ide, AZ Jan Palfijn Gent, Gent, Belgium. Lozel Villadore, Manila Doctors Hospital, Manila, Philippines. Ma. Theresa Diwata Gagan, Lucena City, Quezon, Philippines. Madnieyah Salasa, New Somerset Hospital, Cape Town, South Africa. Maria Katrina Rayos, The Medical City, Pasig City, Philippines. Mariss Catherine Roque, Naga City, Philippines. Mary Crist Jamora, Baguio General Hospital and Medical Center, Baguio City, Philippines. Mihloti Pister Mathonsi, Itshelejuba Hospital, Pongola, South Africa. Nectarios Papavarnavas, Groote Schoor Hospital, University of Cape Town, Cape Town, South Africa. Nirupa Misra, King Dinuzulu Hospital Complex, Ethekwini, KwaZulu-Natal, South Africa. Nomi Aparece, St Luke’s Medical Center, Global City, Taguig City, Philippines. Nomusa Mkhabela, Dundee Provincial Office Hospital, Dundee, South Africa. Nondumiso Sibisi, Vryheid District Hospital, Vryheid, South Africa. Ntombifuthi Bophela, Niemeyer Memorial Hospital, Utrecht KZN, South Africa. Olwethu Ncume, Institute of Public Health, Pietermaritzburg, South Africa. Paul-Emile Claus, Imelda Hospital, Bonheid, Belgium. Sandrine Jacquet, Clinique Saint-Pierre Ottignies, Ottignies, Belgium. Sanele Mngomezulu, Public Hospital, Kwangwanase, South Africa. Sarah Resseler, Ziekenhuis Oost-Limburg, Maas en Kempen, Maaseik, Belgium. Sharon Bucoy, Zamboanga City Medical Center, Zamboanga City, Philippines. Shayne Julieane Morales, Vicente Sotto Memorial Medical Center, Cebu City, Philippines. Shingie Matema, Manguzi Hospital, Manguzi, South Africa. Stephen Rae Fontanilla, Southern Isabela Medical Center, Santiago, Isabela, Philippines. Stijn Jonckheere, Jan Yperman Hospital, Ypres, Belgium. Tasneem Waja (Mukadam), Lenmed Ahmed Kathrada Private Hospital, Johannesburg, South Africa. Tharushni Perumal, Greys Hospital, Pietermaritzburg, South Africa. Theresa Yoro, South Cotabato Provincial Hospital, Koronadal City, South Cotabato, Philippines. Vanessa Aira Rivera, Lorma Medical Center, City of San Fernando, La Union, Philippines. Veerle Westelinck, AZ Sint-Maarten, Mechelen, Belgium. Vita Gasataya, South Cotabato Provincial Hospital, Koronadal City, South Cotabato, Philippines. Welcome Sihle Mbatha, Ngwelezana Tertiary Hospital, Empangeni, South Africa. Xavier Holemans, Grand Hôpital De Charleroi, Charleroi, Belgium. Ysaline Seynaeve, Centre Hospitalier Régional De Verviers, Verviers, Belgium. Zesca Meyer, University of Cape Town, Cape Town, South Africa. Zingisa Mandlana, St Barnabas, Libode, South Africa. Ziningi Ntuli, Manguzi Hospital, Kwangwanase, South Africa.

## Supplementary data

Appendices I–V available as Supplementary data at *JAC-AMR* Online.

## References

[1] Naghavi M, Vollset SE, Ikuta KS et al Global burden of bacterial antimicrobial resistance 1990–2021: a systematic analysis with forecasts to 2050. Lancet 2024; 404: 1199–226. 10.1016/S0140-6736(24)01867-1 39299261 PMC11718157

[2] Poran I, Elbaz M, Turjeman A et al Predicting in-hospital antibiotic use in the medical department: derivation and validation study. Antibiotics (Basel) 2022; 11: 813. 10.3390/antibiotics11060813 35740219 PMC9219723

[3] Beck M, Koll C, Dumpis U et al Identifying patients at high risk for antibiotic treatment following hospital admission: a predictive score to improve antimicrobial stewardship measures. Infection 2025; 53: 1941–52. 10.1007/s15010-025-02525-9 40232662 PMC12460503

[4] Livorsi DJ, Merchant JA, Cho H et al A novel risk-adjusted metric to compare hospitals on their antibiotic prescribing at hospital discharge. Clin Infect Dis 2024; 79: 588–95. 10.1093/cid/ciae224 38658348 PMC11426263

[5] Goodman KE, Baghdadi JD, Magder LS et al Patterns, predictors, and intercenter variability in empiric gram-negative antibiotic use across 928 United States hospitals. Clin Infect Dis 2023; 76: e1224–35. 10.1093/cid/ciac504 35737945 PMC9907550

[6] Tamhankar AJ, Karnik SS, Stålsby Lundborg C. Determinants of antibiotic consumption—development of a model using partial least squares regression based on data from India. Sci Rep 2018; 8: 6421. 10.1038/s41598-018-24883-1 29686420 PMC5913309

[7] Gianino MM, Lenzi J, Bonaudo M et al Predictors and trajectories of antibiotic consumption in 22 EU countries: findings from a time series analysis (2000–2014). PLoS One 2018; 13: e0199436. 10.1371/journal.pone.0199436 29933377 PMC6014649

[8] Bu Q, Wang B, Huang J et al Estimating the use of antibiotics for humans across China. Chemosphere 2016; 144: 1384–90. 10.1016/j.chemosphere.2015.10.010 26492425

[9] Versporten A, Zarb P, Caniaux I et al Antimicrobial consumption and resistance in adult hospital inpatients in 53 countries: results of an internet-based global point prevalence survey. Lancet Glob Health 2018; 6: e619–29. 10.1016/S2214-109X(18)30186-4 29681513

[10] Reilly J, Coignard B, Price L et al The reliability of the McCabe score as a marker of co-morbidity in healthcare-associated infection point prevalence studies. J Infect Prev 2016; 17: 127–9. 10.1177/1757177415617245 28989468 PMC5074213

[11] Verbeke G, Molenberghs G. Linear Mixed Models for Longitudinal Data. Springer New York, 2009.

[12] Russotto A, Gastaldo C, Di Giacomo S et al Health care-associated infections and antimicrobial use: the third point prevalence survey on 42 acute care hospitals in piedmont, Italy, 2022. Am J Infect Control 2025; 53: 855–61. 10.1016/j.ajic.2025.03.019 40113017

[13] Rashid MM, Akhtar Z, Chowdhury S et al Pattern of antibiotic use among hospitalized patients according to WHO Access, Watch, Reserve (AWaRe) classification: findings from a point prevalence survey in Bangladesh. Antibiotics (Basel) 2022; 11: 810. 10.3390/antibiotics11060810 35740216 PMC9220119

[14] Higgins H, Freeman R, Doble A et al Appropriateness of acute-care antibiotic prescriptions for community-acquired infections and surgical antibiotic prophylaxis in England: analysis of 2016 national point prevalence survey data. J Hosp Infect 2023; 142: 115–29. 10.1016/j.jhin.2023.10.006 37858806

[15] Barbadoro P, Dolcini J, Fortunato C et al Point prevalence survey of antibiotic use and healthcare-associated infections in acute care hospitals: a comprehensive report from the Marche Region of Italy. J Hosp Infect 2023; 141: 80–7. 10.1016/j.jhin.2023.07.025 37574019

[16] Sevin T, Daniau C, Alfandari S et al Patterns of antibiotic use in hospital-acquired infections. J Hosp Infect 2021; 114: 104–10. 10.1016/j.jhin.2021.05.008 34052283

[17] Kuster SP, Ruef C, Bollinger AK et al Correlation between case mix index and antibiotic use in hospitals. J Antimicrob Chemother 2008; 62: 837–42. 10.1093/jac/dkn275 18617509

[18] Stenehjem E, Hersh AL, Sheng X et al Antibiotic use in small community hospitals. Clin Infect Dis 2016; 63: 1273–80. 10.1093/cid/ciw588 27694483

[19] Piekiełko P, Mucha A, Stawowczyk E et al Peripheral intravenous therapy in internal medicine department—antibiotics and other drugs’ consumption and characteristics of vascular access devices in 2-year observation study. Antibiotics 2024; 13: 664. 10.3390/antibiotics13070664 39061346 PMC11274068

[20] Wallis MC, McGrail M, Webster J et al Risk factors for peripheral intravenous catheter failure: a multivariate analysis of data from a randomized controlled trial. Infect Control Hosp Epidemiol 2014; 35: 63–8. 10.1086/674398 24334800

[21] Belgian Society for Infectiology and Clinical Microbiology (SBIMC–BVIKM) . Launch of IGGI 2.0—Updated Guidelines for Infection Prevention Now Available. https://sbimc-bvikm.be/en/iggi.

[22] For a Healthy Belgium . Hospital Outbreak Support Team. https://www.healthybelgium.be/en/key-data-in-healthcare/covid-19-en/quality/hospital-outbreak-support-team-en.

[23] Belgian Antibiotic Policy Coordination Committee (BAPCOC) . HOST Project Annual Report 2023. Belgian Federal Public Service Health, Food Chain Safety and Environment, 2023.

[24] Oliveira I, Rego C, Semedo G et al Systematic review on the impact of guidelines adherence on antibiotic prescription in respiratory infections. Antibiotics (Basel) 2020; 9: 546. 10.3390/antibiotics9090546 32867122 PMC7557871

[25] Héquet D, Kessler S, Rettenmund G et al Healthcare-associated infections and antibiotic use in long-term care residents from two geographical regions in Switzerland. J Hosp Infect 2021; 117: 172–8. 10.1016/j.jhin.2021.08.018 34428504

[26] Virtanen M, Terho K, Oksanen T et al Patients with infectious diseases, overcrowding, and health in hospital staff. Arch Intern Med 2011; 171: 1296–8. 10.1001/archinternmed.2011.313 21788550

[27] Christensen I, Haug JB, Berild D et al Factors affecting antibiotic prescription among hospital physicians in a low-antimicrobial-resistance country: a qualitative study. Antibiotics (Basel) 2022; 11: 98. 10.3390/antibiotics11010098 35052975 PMC8773165

[28] Ruiz-Garbajosa P, Cantón R. COVID-19: impact on prescribing and antimicrobial resistance. Rev Esp Quimioter 2021; 34 Suppl 1: 63–8. 10.37201/req/s01.19.2021 34598431 PMC8683018

[29] Haug JB, Berild D, Walberg M et al Hospital- and patient-related factors associated with differences in hospital antibiotic use: analysis of national surveillance results. Antimicrob Resist Infect Control 2014; 3: 40. 10.1186/s13756-014-0040-5 25598971 PMC4296539

[30] Yan K, Xue M, Ye D et al Antibiotic prescribing practices in secondary and tertiary hospitals in Shaanxi province, western China, 2013–2015. PLoS One 2018; 13: e0207229. 10.1371/journal.pone.0207229 30540753 PMC6291232

[31] Ogunleye OO, Oyawole MR, Odunuga PT et al A multicentre point prevalence study of antibiotics utilization in hospitalized patients in an urban secondary and a tertiary healthcare facilities in Nigeria: findings and implications. Expert Rev Anti Infect Ther 2022; 20: 297–306. 10.1080/14787210.2021.1941870 34128756

[32] Chen R, Li J, Wang C et al Global antibiotic prescription practices in hospitals and associated factors: a systematic review and meta-analysis. J Glob Health 2025; 15: 04023. 10.7189/jogh.15.04023 39883879 PMC11781807

[33] Zay Ya K, Win PTN, Bielicki J et al Association between antimicrobial stewardship programs and antibiotic use globally: a systematic review and meta-analysis. JAMA Netw Open 2023; 6: e2253806. 10.1001/jamanetworkopen.2022.53806 36757700 PMC9912134

[34] Federal Public Service Health Food Chain Safety and Environment . https://www.healthybelgium.be/en/key-data-in-healthcare/general-hospitals/organisation-en.

[35] Department of Health . Philippine Health Facility Development Plan 2020–2040. Manila, Philippines: Department of Health, 2020. https://bit.ly/PHFDP2020_2040.

[36] Dell A, Kahn D. Geographical maldistribution of surgical resources in South Africa: a review of the number of hospitals, hospital beds and surgical beds. S Afr Med J 2017; 107: 1099–105. 10.7196/SAMJ.2017.v107i12.12539 29262964

