# Supplementary material for: Identifying hospital-level predictors for antibiotic use: a Global Point Prevalence Survey study among Belgian, Philippine and South African hospitals
Source: JAC Antimicrob Resist. 2026 Apr 3;8(2):dlag042. doi: 10.1093/jacamr/dlag042 (PMC13048902; doi:10.1093/jacamr/dlag042)
Supplement: dlag042_Supplementary_Data [file dlag042_supplementary_data.docx]

**Supplementary Materials**

[**Appendix I –** Protocol of the basic inpatient module and healthcare-associated infection **2**](#_Toc212739379)

[**Appendix II –** Data collection forms of the basic inpatient module and healthcare-associated infection (HAI) module of the Global Point Prevalence Survey (Global-PPS) **24**](#_Toc212739391)

[**Appendix III –** Calculation of the predictors of the Global-PPS **34**](#_Toc212739392)

[**Appendix IV –** Estimates of the linear mixed model to predict hospital-level prevalence of antibiotics from Global-PPS data **37**](#_Toc212739393)

[**Appendix V –** Results of the Type III ANOVA tests from the linear mixed model to predict hospital-level prevalence of antibiotics from Global-PPS data **38**](#_Toc212739394)

## **Appendix I –** Protocol of the basic inpatient module and healthcare-associated infection (HAI) module of the Global Point Prevalence Survey (Global-PPS)

| **Global Point Prevalence Survey of**  **Antimicrobial Consumption and Resistance**  **(2023 GLOBAL-PPS)**  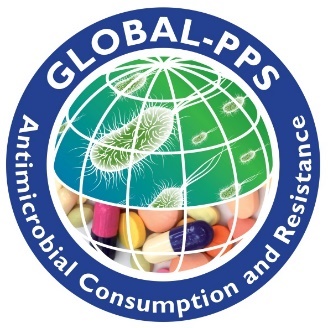  URL : [www.Global-PPS.com](http://www.Global-PPS.com)  **Protocol to collect inpatient prescribing data**  *- version April 2023 -*  **Lead Investigator:** Erika Vlieghe (University Hospital of Antwerp, Belgium)  **Coordinating Centre & Technical Support:** Ann Versporten, Ines Pauwels, Annelies Boven, Nico Drapier, Jimmy Keustermans, Anna Ivanova, Laboratory of Medical Microbiology, University of Antwerp, Antwerp, Belgium.  **Private Funding Authority:** bioMérieux  **Timeline:** three surveys will be available each year to allow investigating seasonal variation:  **Data should be “collected” within 3 predefined time frames/year**:   - January-April - May-August - September-December   Herewith, data collection should be finished within the period it was started. Data entry into the Global-PPS tool is possible at least up to one month after the predefined period.  **Any hospital from any country worldwide is welcome to participate.**  **Hospitals can choose one or more appropriate periods of participation.**  **April 2023 version of protocol:**   - Addition of a fully integrated outpatient module - Minor changes to the inpatient module (e.g. reason missed doses: addition “declined”)   **February 2022 version of protocol : changes with respect to the January 2021 version of the protocol:**   - Addition of intramuscular (IM) administration of antimicrobials next to intravenous (IV) administration. - Addition of optional variable “missed doses” and “reason of missed doses” in the basic patient form. - More in-depth clarification on how to include patients on surgical prophylaxis. - Addition of ‘stool’ in list of sample types for ‘cultures sent to the lab’. - Addition of ‘IS-AMW (isolation ward)’ and ‘AHDU (High Dependency Unit)’ in ward form.   **January 2021 version of the protocol : changes with respect to the May 2020 version of the protocol:**   - Clarification ‘Non-invasive positive and negative mechanical ventilation (CPAP, BiPAP, CNEP,...)’ in the denominator and numerator.   **May 2020 version of the protocol : changes with respect to February 2020 version of the protocol:**   - addition of ‘COVID-19’ in Appendix II – diagnostic codes of the data collection templates p 7. - addition of ‘Non-invasive mechanical ventilation (CPAP, BiPAP, CNEP)’ in the denominator (ward form, see data collection templates p1) and numerator (HAI patient form, see data collection templates p4). - update of the antimicrobial list with Remdesivir. All other drugs currently used to treat COVID-19 were already on our antimicrobial list available at <https://www.global-pps.com/documents/>   **Contents**  [**BACKGROUND - AIMS** 4](#_Toc212729864)  [**GLOBAL-PPS Protocol Specifics** 6](#_Toc212729865)  [**WebPPS - STEP BY STEP PROCEDURE** 11](#_Toc212729866)  [**DATA COLLECTION FORMS** 14](#_Toc212729867)   - [The WARD form 14](#_Toc212729868) - [The PATIENT form 16](#_Toc212729869) - [The HAI-PATIENT form: Optional HAI module 21](#_Toc212729870) - [HOSPITAL PROFILE: Optional data to collect at hospital level 21](#_Toc212729871)   [**EXPORT YOUR DATA** 22](#_Toc212729872)  [**VALIDATION PROCESS** 22](#_Toc212729873)  [**FEEDBACK** 23](#_Toc212729874)   \| **THE DATA COLLECTION FORMS AND APPENDICES ARE AVAILABLE AS A SEPARATE DOCUMENT TO THIS PROTOCOL:**  Forms:   - Ward form - Patient form - HAI-patient form - additional variables for HAI module (optional) - Hospital profile (optional)   Appendices:   - Appendix I: Combination Anti-Infective Agents - Appendix II : Diagnostic Codes - Appendix III : Type Of Indication - Appendx IV: List Of Micro-Organisms By Resistance Type - Print one ward form for each different ward. - Print one patient form **for each patient on antimicrobials.** - Print one patient form “additional variables for HAI module” **for each patient on antimicrobials.** - No need to fill in the basic and the HAI patient form for patients not on antimicrobials! \| \| --- \|  BACKGROUND - AIMS The Global Point Prevalence Survey (Global-PPS or G-PPS) is a simple, freely available web-based tool to measure and monitor antimicrobial prescribing and resistance in hospitals worldwide ([www.global-pps.com](http://www.global-pps.com)). The Global-PPS has established a global network of hospitals conducting point prevalence surveys and provides quantifiable measures to assess and compare quantity and quality of antimicrobial prescribing, prevalence of healthcare-associated infections (HAI) and resistance in hospitalized adults, children and neonates worldwide  The Global-PPS was first piloted in 2014, with worldwide studies conducted in 2015^[[1]](#footnote-1)^ and 2017. Since 2018, three survey a year are available. The Global-PPS is coordinated at the University of Antwerp, Belgium and sponsored through an unrestricted grant given to them annually by bioMérieux.  Over 1300 institutions from more than 90 different countries worldwide have participated at least once in the Global-PPS. As a result, the database now includes more than 500,000 patients allowing benchmarking of hospitals from similar settings (hospital types or departments such as intensive care, hemato-oncology, internal medicine and surgery), in the same country, region and time periods.  We observed many interesting findings following these Global-PPS. For instance, penicillin with β-lactamase inhibitors, third-generation cephalosporins, and fluoroquinolones were the three most prescribed antimicrobials worldwide, reflecting high rates of prescribing of mainly broad-spectrum antibiotics. Carbapenems were most frequently prescribed in Latin America and West- and Central Asia. Most of these antibiotics were prescribed for empirical use, illustrating the lack of diagnostics to document infections. Local antibiotic guidelines were missing for 7,050 (19.2%) of the 36,792 antibiotic prescriptions. Guideline compliance was 77.4%. Finally, one of the main problems of prolonged antibiotic use was peri-operative prophylaxis (overall, duration of antibiotic peri-operative prophylaxis was more than one day in about 80% of surgical patients in low-and middle-income countries (LMIC)).^2^  The Global-PPS complies with the WHO global action plan on antimicrobial resistance as requested by the Health Assembly in resolution WHA67.25, May 2014^[[2]](#footnote-2)^. The goal of the global action plan was to ensure continuity of successful treatment and prevention of infectious diseases with effective and safe medicines that are quality-assured, used in a responsible way, and accessible to all who need them. The Global-PPS responds to its strategic objectives in order to meet the goal: (1) improve awareness and understanding of antimicrobial resistance; (2) strengthen knowledge through surveillance and research; (3) reduce the incidence of infection; (4) optimize the use of antimicrobial agents; and (5) ensure sustainable investment in countering antimicrobial resistance. As such, the Global-PPS has been instrumental in informing stewardship activities in many participating hospitals^[[3]](#footnote-3)^. By providing all hospitals with a personalised feedback report, the Global-PPS allows local teams to identify targets for antimicrobial stewardship without the need to invest time and resources in complex data analyses.  **Main aims of the Global-PPS**   - Survey performance indicators and **identify targets for quality improvement** **of antimicrobial prescribing and HAI** (e.g. duration of peri-operative prophylaxis; compliance with local hospital guidelines; prevalence of HAI amongst invasive procedures) **(identify burden!)** - Helps in designing hospital interventions that aim at **promoting prudent use of antimicrobials as well as the prevention of HAIs (change practice!)** - Allows to assess the effectiveness of such interventions, through repeated PPS **(measure impact!)**   The Global-PPS tool supports the concept of “simplicity and feasibility”. Moreover, detailed information on patients, antimicrobial use and indicators with respect to HAI is only requested from patients receiving at least one antimicrobial on the day of the PPS; and denominator data is collected separately at ward level. In practice, this means that for a 400-bed hospital (and 100% bed occupancy) and an estimated antimicrobial prevalence of 40%, detailed information only needs to be collected for 160 patients (and not for 400 patients). In this way, the Global-PPS provides an easy hands-on tool that can be repeated easily to support stewardship programs.  **This tool has illustrated many core benefits:**   - The web-based tool is easy to use, requiring minimal training for data entry; - The hospital is able to download a real-time one-point and longitudinal feedback report which can be used for local communications and presentations; - There is evidence of consistency and reproducibility with the data entry using this tool; - Participation in the survey has encouraged thorough engagement and feedback, enhancing communication between prescribers and the local infection community; - The Global-PPS enables sharing best practices and raises awareness of inappropriate antimicrobial prescribing with broad adaptability and suitability for a range of health care resource settings.  GLOBAL-PPS Protocol Specifics**Time planning for the PPS** Any participating hospital must complete the Point Prevalence Survey (PPS) within a maximum of *4 consecutive weeks* from the time when the hospital starts data collection.  **Data should be collected within three proposed time frames a year:**   \| - - January–April   - May-August   - September-December \| \| --- \|  **Departments involved** The departments are grouped into medical and surgical adult departments, adult intensive care units, paediatric and neonatal departments (see page 12: “Prepare hospital department list” and in the data collection forms (Page1, ward form)).  All wards (or units/departments) within the participating hospital should preferably be included if you participate for the very first time. Bigger hospitals who have participated to one or more of the previous surveys can ***participate with a number of wards (subsample)*** of the hospital, but they need to include all wards for a certain “main” activity (medical, surgical, ICU): for example include all adult ICU wards, all adult surgical wards, all adult medical wards or all child medical wards. This is needed to allow the collection of sufficient data by “activity” and subsequently the calculation of valid rates by activity. For smaller hospitals (± <200 to 250 beds), it remains advisable to survey the whole hospital!  Each ward included into the survey has to be surveyed **only once on a single day** in order to calculate correctly the denominator (number of admitted patients). However, different wards can be surveyed on different days.  Each hospital shall decide on which days data collection shall take place (depends on the size of the hospital and own organization) as long as it is within the stipulated time frame. **Inclusion criteria**  **All inpatients** admitted on a ward (excluding day admissions such as endoscopy or renal units) **at 8 o’clock** in the morning on the day of survey count in the denominator. All inpatients “**on** **antimicrobial agents” at 8 o’clock** in the morning on the day of survey are to be included in the numerator (i.e., a patient form is to be filled in for these patients only).   - Definition of “**on** **antimicrobial agents”**: - A patient receiving an antibiotic e.g. every 48 hours but not receiving this antibiotic on the survey day must be included = ongoing antimicrobial treatment. - An antibiotic prescribed at one o’clock (during the ward round or when results become available or for surgical prophylaxis) in the afternoon on the day of the survey must **not** be included (not active or ongoing at 8 o’clock in the morning). - Include **new-born healthy children** on a maternity ward. Encode this ward as a supplementary neonatal medical ward (NMW).   **How to encode antibiotics for surgical prophylaxis (SP) ?**  **Surgical Wards** (PSW, ASW) **should be surveyed** **on the day following the day when most elective surgical interventions usually take place or have been planned**; in order to capture information about surgical prophylaxis in the previous 24 hours. (e.g. If a surgical ward mainly has surgeries planned on Tuesdays, then the ward should be surveyed on Wednesday).  The administration of antibiotics for SP **should be** **checked in the previous 24 hours** in order to encode the duration of prophylaxis as either one dose, one day (=multiple doses given in one day) or >1 day. This means that patients admitted on the ward at 8am on the day of the PPS, and who received SP ‘**before**’ 8am on the day of the PPS, will be included in the survey. For those patients, a patient data collection form needs to be completed to capture information on the antibiotic(s) prescribed for SP!   1. **Include all prescribed antimicrobials not stopped at 8am or initiated on the dot at 8am** for all therapeutic or prophylactic indications including surgical prophylaxis (SP still ongoing on the day of the PPS = SP>1 day (SP3)). 2. **Include also those antimicrobials prescribed for SP and administered the day before the PPS** (=the day most interventions were planned) to allow capturing whether the SP was one dose (SP1) or multiple doses and stopped the day before the PPS (SP2), in addition to SP>1 day (SP3).   **All these antimicrobials need to be added on the patient form.**  For more detailed examples of how to encode antimicrobials for SP, see also table below**:**  Patients who receive surgical prophylaxis after 8am on the day of the PPS are NOT included in the survey (see exclusion criteria).  Include patients on surgical prophylaxis marked in green:  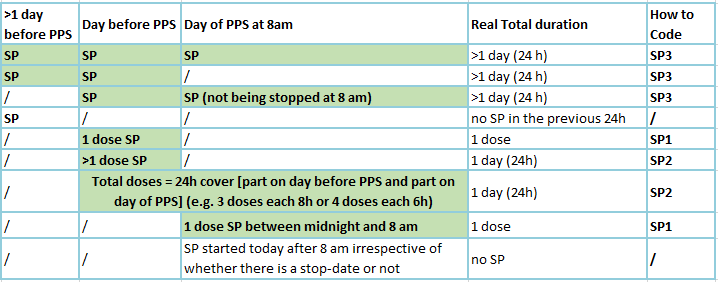  **Intensive Care and Medical wards** (all other wards) can be surveyed at any weekday except on weekends or bank holidays. **Exclusion criteria**   - Exclude day hospitalizations and outpatients. These are defined as ambulatory care patients. So, data from “day” surgery and “day” hospital units should be excluded from the survey. - Exclude patients admitted after 8 o’clock on the day of the survey (even though these would be present by the time the survey is carried out). All patients/wards falling in the exclusion criteria must be excluded from BOTH the numerator and denominator data.  **Denominator data**   - ***Total number of admitted inpatients* at 8 am** of the ward surveyed. Do not collect data from patients discharged before 8 o’clock and/or patients admitted after that time. In the Ward Form, the denominator refers to the total number of eligible admitted patients on the ward at 8 o’clock. - ***Total number of available beds* attributed to inpatients at 8 am** of the ward surveyed. This means the number of total inpatient beds at the time of the survey. Total number of beds includes occupied beds + empty beds.  N beds should always be ≥ N admitted inpatients present at 8 o’clock (=occupying a bed) on the survey day. - For the optional HAI-module add also the “***Total number of admitted inpatients having an invasive device inserted at 8 am*** on the survey day”. Denominators for 6 different devices need to be completed (see data collection ‘Ward form’).  **Included antimicrobial agents**  - ***Antibacterials for systemic use***: J01 - ***Antimycotics and antifungals for systemic use***: J02 & D01BA (including griseofulvin and terbinafine) - ***Drugs for treatment of tuberculosis***: J04A (these are the antibiotics as well as all other drugs to treat tuberculosis) - ***Antibiotics used as intestinal anti-infectives***: A07AA - ***Antiprotozoals used as antibacterial agents****,* ***nitroimidazole derivatives:*** P01AB - ***Antivirals for systemic use***: J05 - ***Antimalarials***: P01B   **Antimicrobials for topical use are excluded from the survey**.  The Global-PPS tool provides the list of all antimicrobials to be surveyed according to the WHO ATC classification^[[4]](#footnote-4)^. The antimicrobial list (excel) is available at [www.global-pps.com/documents](http://www.global-pps.com/documents/). The file contains all substances with their route of administration. The file also provides information on the antimicrobial (sub)classes. In case a drug is not in the provided list, contact [Global-PPS@uantwerpen.be](mailto:Global-PPS@uantwerpen.be) **Multidisciplinary team** The hospitals are invited to create a multidisciplinary team of colleagues familiar with reading patient notes and having adequate knowledge on local guidelines (e.g., infectious disease specialists, microbiologists, pharmacists, infection control specialists, nurses or other healthcare professionals). A ***local administrator*** has to be assigned and he/she will be the main contact person for the Global-PPS Coordinating Centre & Technical Support team at the University of Antwerp, Belgium.  The local administrator is responsible for:   - the online registration of the hospital(s), - entering patient-specific data into the *Global-PPS tool*, - the data validation and - the production of the local feedback reports.   Extra hospital users may, however, be registered within the Global-PPS tool in order to help the local administrator with data entry (see IT manual – add supplementary hospital users, for more information with respect to centralized data entry by a network coordinator; available at [www.global-pps.com/documents](http://www.global-pps.com/documents)). **Data Privacy**  A sequence number will be assigned to each *hospital* after registration in the Global-PPS tool. Hospital names will never be revealed in any report or publication without approval from the participant (e.g. for peer-reviewed articles).  *Patients* are completely anonymised in the Global-PPS tool. Every patient record will be given a unique not identifiable survey number. This number is automatically generated by the computer program, based on several internal codes. This number identifies the patient uniquely in the *GLOBAL-PPS* database. For more information, consult the data privacy excerpt (contact [global-pps@uantwerpen.be](mailto:global-pps@uantwerpen.be)). **Data ownership**  - Data are the property of the respective hospital. - The Global-PPS Coordinating Centre & Technical Support team at the University of Antwerp, Belgium is guardian of the data within the database; - will analyse the data and generate reports. These analyses and reports are property of the Global-PPS; - encourages country- and/or region-specific analyses.   For more information, consult the data privacy excerpt (contact [global-pps@uantwerpen.be](mailto:global-pps@uantwerpen.be)).  **Ethical approval** For approval by ethical committee & privacy legislation requirements, the Global-PPS Coordinating Centre & Technical Support team can provide, on request, a letter that can be submitted to hospitals ethical committees (contact [Global-PPS@uantwerpen.be](mailto:Global-PPS@uantwerpen.be)).  **Technical support** The Global-PPS Coordinating Centre & Technical Support team at the University of Antwerp provides a "help desk" for software or any other issues encountered and/or questions during the data collection and data entering ([Global-PPS@uantwerpen.be](mailto:Global-PPS@uantwerpen.be)). They will continually be available for general queries about the project.  The Global-PPS tool <https://app.globalpps.uantwerpen.be/globalpps_webpps/> offers:   - 1. internal checks in order to avoid invalid or erroneous figures (e.g. for out-of-range values)   2. boxes popping up in order to guide you to fill out a field   3. help functions which provides supplementary information on each screen (on top, left side of screen)   4. Help pages, IT manual, “FAQ” list.   Web page layout for the forms will be similar to the paper version.  Regular backups of the database will guarantee the integrity of data.  The format to export data is Microsoft Excel^®^.  The software and database are hosted on a server at the University of Antwerp in Belgium, Europe. The Global-PPS Coordinating Centre & Technical Support team can provide more details on ensured data protection and safeguarding (contact [global-PPS@uantwerpen.be](mailto:global-PPS@uantwerpen.be)) .  **Publication policy**  The Global-PPS Coordinating Centre & Technical Support team should look for opportunities for dissemination and encourage country-specific analyses. For publications at national or regional level, **participants need to comply with the publication strategy** as designed by the Global-PPS Coordinating Centre & Technical Support team. The publication strategy will guide you on how to proceed. The publication policy is available at [www.global-pps.com/documents](http://www.global-pps.com/documents).  **The optional Healthcare-Associated Infection (HAI) module**This module enables to survey healthcare-associated infections in more detail, allowing analyses of additional quality indicators. The main focus lies on the presence of invasive devices.The module is optional, but once a participating hospital decides to sign up for this module, an HAI form needs to be completed for each patient receiving an antimicrobial on the day of the PPS. This additional patient form includes 4 mandatory variables and 3 optional variables. All variables referring to a date are also optional to complete (see this protocol p 20; and data collection templates p 4).The additional denominators, collected at ward level, are available on the ward form and are mandatory to be completed if a hospital chooses to include the optional HAI module (see data collection templates, ward form, page 1). The Global-PPS collects information about the indication for antimicrobial prescriptions. This is to be interpreted as what the clinician aims at treating. To obtain this information the investigator should be looking at all [medical, nursing and drug prescription chart] patient records. If the information available is not sufficient, surveyor/s may request additional information from nurses, pharmacists or doctors caring for the patient. Searching for information from other sources such a laboratory computer systems, phoning laboratories *etc.,* is not required.  **At no point shall there be any discussion about the appropriateness (or lack thereof) of the prescribed medication.** **The ward staff MUST NOT feel evaluated at the individual level**. **WebPPS - STEP BY STEP PROCEDURE** All the following steps must be completed before entry of any patient data.  Before the hospital submits any patient data to the WebPPS, the following steps must be completed:  ***Hospitals who have participated in the Global-PPS previously need to login with their existing password which is linked to an email address! Only then, you will be able to activate a new survey using the same hospital number as the one which was attributed to you previously. Only then, you will be able to download a longitudinal feedback including results on previous surveys you conducted.***  ***If you lost your login, or if you are a new participant/member for a hospital who participated in previously, contact Ann/Ines at*** [***global-pps@uantwerpen.be***](mailto:global-pps@uantwerpen.be)  ***Applicable for hospitals who participate for the very first time:***   1. **Register yourself and the hospital on the Global-PPS website:**   First-time participants **for “hospitals who never participated” to the Global-PPS** need to register themselves on https://app.globalpps.uantwerpen.be:8443/globalpps_webpps/register. They will receive an email to confirm their email address (in order to avoid spam registration). Thereafter they can login on the Global-PPS tool (do not forget to tick the captcha): https://app.globalpps.uantwerpen.be:8443/globalpps_webpps/login.  Thereafter the participant needs to **register the hospital(s) on the Global-PPS tool** (see IT manual available online: [www.global-pps.com/documents](http://www.global-pps.com/documents)). The person who registers the hospital for the very first time will be the local administrator. He/she has access to all functionalities of the program. Included hospital types^[[5]](#footnote-5)^ are primary, secondary, tertiary, specialized, infectious diseases and paediatric hospitals.  The local administrator can **add extra users** in order to give other person(s) the rights to enter data as well for his hospital (see IT manual at [www.global-pps.com/documents](http://www.global-pps.com/documents/)).  ***Applicable for hospitals who participated at least once to any of the previous Global-PPS:***  Second and subsequent participations to GLOBAL-PPS when the hospital has already been registered with the GLOBAL-PPS:   - The already registered and known participant needs to use their existing login (username) and password ! If this information has been lost, contact [Global-PPS@uantwerpen.be](mailto:Global-PPS@uantwerpen.be) - New participants belonging to a hospital who participated before need to contact the local administrator of the hospital. If the local administrator is not working any more at the hospital, please contact Ann or Ines ([Global-PPS@uantwerpen.be](mailto:Global-PPS@uantwerpen.be)). They will do the necessary to link you up with the existing hospital. Important, do not create a new hospital (with another ID number) as you will then not be able to download a longitudinal feedback report!  1. **Prepare the hospital department list**   ***Hospitals who participated before need to review the hospital department list and make necessary updates if needed (e.g. adding a new department, change the name of the department).***  **New participating hospitals:**  After login, one first needs to define ALL the hospital’s wards (units/departments) as these will afterwards appear in the drop down lists when entering patient information.  The definition of a department follows a hierarchical structure. For each department, the following information is needed:   - The **NAME** of the department. This field is mandatory and identifies the department uniquely in the database. This uniquely defined name will be used in the drop down lists in the *Global-PPS tool*. **All** inpatient adult, paediatric and neonatal departments must be included (i.e., no hospital sampling). - Code and description = optional. It allows you to describe the department name in more detail if needed - Department **GROUP** (Adult medical, surgical or intensive care ward, paediatric ward) - Specify **TYPE** of department/ward. Mandatory field. Choose, if available in the hospital, between the following specialties:  \| - 15 different **Adult medical wards (AMW)**   **AMW** (General or mixed specialties)  **HO-AMW** (Haematology-Oncology)  **T-AMW** (Transplant (BMT/solid))  **P-AMW** (Pneumology)  **CAR-AMW** (Cardiology)  **NEU-AMW**(Neurology)  **REN-AMW** (Nephrology)  **ID-AMW** (Infectious Disease)  **DB-AMW** (Dermatology-burn wards)  **PSY-AMW** (Psychiatry)  **REH-AMW** (Rehabilitation)  **GER-AMW** (Geriatrics)  **LTC-AMW** (Long-Term care)  **OBG-AMW** (gynaecology-obstetrics)  **IS-AMW** (Isolation ward, e.g. COVID patients) \| - 9 different **Adult surgical wards (ASW)**   **ASW** (General or mixed specialties)  **DIG-ASW** (Digestive tract surgery)  **ORT-ASW** (Orthopaedics-Trauma surg.)  **URO-ASW** (Urological surg.)  **CV-ASW** (Cardio-Vascular surg.)  **NEU-ASW** (Neurosurgery)  **ONCO-ASW** (Oncology-cancer surg.)  **PLAS-ASW** (Plastic, reconstructive surg.)  **ENT-ASW** (Ear-nose-throat surg.)   - 5 different **Adult Intensive Care Units (AICU)**   **AICU** (General or mixed specialties)  **MED-AICU** (Medical AICU)  **SUR-AICU** (Surgical AICU)  **CAR-AICU** (Cardiac AICU)  **AHDU** (High Dependency Unit) \| \| --- \| --- \| \| - 6 **Paediatric wards**   **PMW** (Paediatric Medical Ward)  **HO-PMW** (Haematology-Oncology PMW)  **T-PMW** (Transplant (BMT/Solid) PMW)  **PSW** (Paediatric Surgical Ward)  **PICU** (Paediatric Intensive Care Unit)  **ID-PMW** (Infectious Disease PMW) \| - 2 **neonatal wards**   **NMW** (Neonatal Medical Ward)  **NICU** (Neonatal Intensive Care Unit \|   ***Examples of a difficult case:***   - A mixed PICU and NICU department should be split up if the number of NICU and PICU assigned beds for the mixed NICU-PICU ward is reasonably stable. Thus, define the ward into 2 different wards (a PICU and NICU ward). - The **ACTIVITY** for a department (**Medicine, Surgery, Intensive Care**) is automatically assigned by the software based on the selected type of department. This is the **“MAIN” attributed activity** of a certain department. This main activity can never be changed or deactivated. Besides the “main” activity of a department, it is still possible to define the department as a mixed department on the day of the survey (if on the day some of the patients are from a different activity). This has to be done when entering the denominator survey data (see data collection templates, ward form, page 1).   The different departments are entered ***manually*** in the *Global-PPS tool*.   1. **Select the appropriate survey**   Select the survey online: go to Surveys/available surveys and **register for the correct survey according the appropriate time frame (Jan-Apr; May-Aug; Sept-Dec). Select the module(s) you want to participate in.** Thereafter go to Surveys/subscribed and click on the appropriate survey to activate the survey for data-entry.   1. **Complete denominators for ALL wards surveyed**   See data collection forms – “Ward form, page 1”  One needs to complete denominator data for each ward surveyed “**before”** entering the first patient. Collected denominators are:   - Total number of admitted patients (count of the total patients on at least one antimicrobial **+** all patients NOT on antimicrobials) - Total number of beds - Total number of admitted patients with an inserted invasive device (for the optional HAI module only)      1. **Enter patient data**   ONLY after all of the steps mentioned above have been carried out (1 to 4), one can start entering patient data*.*  **See IT manual and tutorial video’s** available on the data entry environment to proceed with online data entry step by step : <https://app.globalpps.uantwerpen.be/globalpps_webpps/> **DATA COLLECTION FORMS** To facilitate the data collection, print one single paper ward form for each ward under surveillance and a number of ‘patient forms’ depending on the number of patients on the ward who are on antimicrobial therapy. Print also the corresponding appendices. The data collection forms correspond to the online web forms.   - Ward form - Patient form - The HAI-Patient form to collect additional variables for the optional HAI module   After data collection for each ward, attach to the ward form all the individual patient forms (those patients with antimicrobial treatment, including prophylaxis) and HAI-patient forms. The WARD form **Date of survey** – The date the department/ward is surveyed: dd/mm/yyyy.  **Auditor code** – Code, initials or else of the person completing the form. The code can be used to track possible bias linked to the auditor.  **Hospital name**– The hospital name.  **Ward name**– “Unique” name of the ward.  **Ward Type** – The official ward type split up for medical and surgical adult wards, adult intensive care units, paediatric and neonatal wards (e.g. if a surgical ward is taking overflow from Medicine it is still to be listed as Surgery). The complete list is available in the data collection templates, page 1.  **Mixed ward** – If the department is mixed, tick the Yes case, if not, tick the No case.  **A special case: a mixed ward with mixed activity:** In hospitals with shared beds and mixed wards the denominator is difficult to measure. In fact, there are two separate issues: i) wards which occasionally take patients from a different specialty than their ‘official’ department and ii) permanently mixed wards with no available data on number of beds for each speciality.  For that reason, during data entry and when necessary, it is possible to define a ward as a mixed ward. In this case, the different ***activities*** encountered at the day of survey should be specified, in addition to the encountered denominators : the total number of admitted patients; total number of beds and total number of patients with invasive device (HAI optional module) for each activity according to the defined activity. If needed, the auditor should ask a healthcare worker if any patients belonging to another department are present, before starting the ward survey.  A mixed department will be defined based on the activity level.  **Activity** –Select all the encountered activities (Medicine, Surgery, Intensive Care) based on the activity on the day of the survey. A supplementary ward activity may be defined besides the “main” activity (main activity is automatically attributed by the Global-PPS tool during the department list preparation (see “*Prepare the hospital department list.*”). (e.g. if a surgical ward is taking overflow from Medicine it is still to be listed as Surgery: Define here a mixed department by ticking the box medicine beside surgery as main activity.  **Denominators -**   1. **Total number (N) of admitted inpatients in the department at 8 am** on the day of the PPS should be entered in the column of the corresponding activity. In case of mixed department, the number of admitted patients corresponding to each of the encountered activities should be entered. Reminder: Do not count patients discharged before 8 o’clock and/or patients with admission planned after that time.   An admitted inpatient is a patient occupying a bed at 8am on the day of the PPS. It **includes all patients on antimicrobials + all patients NOT on antimicrobials at 8am on the day of the PPS**.   1. **Total number (N) of “available” beds for inpatients in the department at 8 am** on the day of the PPS. In case of mixed department, fill the total number of beds corresponding to each of the encountered activities.   Total number of beds = total beds in ward (=**occupied + empty beds**). N beds is always ≥ N inpatients present at 8 am.   1. **Total number (N) of admitted inpatients with an “invasive device” present at 8 am** on the day of the PPS **(optional HAI module only).** In case of a mixed department, the number of admitted inpatients with an inserted device should correspond to each of the encountered activity.   Definition of **“*an (inserted) invasive device*”** :   - - **Count also** patients with a device with intermittent insertion, and “accidentally or intentionally” removed before 8am but planned re-insertion after 8am   - **Do not count** patients with a device with planned (intentional) removal before 8 am and whereby no re-insertion is planned after 8am.   Definitions of **the 6 different invasive devices are provided on p 20.**  **Examples of challenging denominator attribution:**   - *Mixed department surgical-medical*: fill in N surgical and medical patients and; when not exactly defined following formal hospital listing, distribute surgical and medical empty beds proportionally (or evenly) in N surgical and medical beds. - *An overbooked department*: provide information of the “actual real situation” on the day of the PPS providing e.g. the total number of patients admitted and total N beds on the day of the survey. - *There are more patients than beds on the ward*: Adapt the number of beds according to the number of inpatients present at 8 am on the day of the PPS. As such, N beds = N inpatients present at 8 am on the day of the PPS.   ***Complete denominator data (=N patients and N beds; and optionally for the HAI module N patients with an invasive device) for wards surveyed “before” entering the first patient.*** The PATIENT form ***Data collected at patient level***  **Ward (Name/code)**– This is the unique **name** of the ward studied. This name is selected using the drop down list in the Global-PPS tool, as this department name has been defined during the preparation of the hospital department list (see step “prepare the hospital department list”, page 12)  **Activity** – When the ward is a **mixed department**, the activity to which the patient belongs must be specified (M: Medicine, S: Surgery, ICU: Intensive Care).  **Full Patient Identifier** – This is a unique number allowing local tracing to the patient level for eventual clarifications. (For example the clinical record/note number, hospital number, etc.) This information will not (and cannot) be reported or submitted in the *Global-PPS* database.  **Survey Number** – It’s a unique non identifiable number generated by the Global-PPS tool for each patient record. Please ensure that the person entering the data online ***writes down this number immediately when it is generated by the tool as it will not be displayed again***. This number identifies uniquely the patient in the *Global-PPS* database.  **Age** – Three fields, one for the year, one for the month and one for the days are available. ***“Only one“ of these fields needs to be completed as follows:***   - If less than 30 days old, write the exact numbers of days completed. - For patients older than 1 month and younger than 2 years fill in month field. (e.g. 19 months) - If the patient is at least 2 years old then only the year field is to be inputted.   **Current weight** – Write the current weight in Kg with one decimal number. *Optional field.*  **Only for neonates** *(optional fields):*  **Gestational age -** Write the “completed” number of gestational weeks without the number of days. E.g. for 33^+4^ write only 33.  **Birth weight** – Write the birth weight in Kg with one decimal number.  **Sex** – M (Male), F (Female), U (Unknown)  **Treatment based on biomarker data or white blood cell count (WBC)** – Tick ‘Yes’ or ‘No’. It refers to whether or not biomarker results close to the start of the antibiotic treatment are used to initiate the treatment. If yes, next lines should also be completed with 4 possible answers (report the most relevant one):   - **CRP** = in case the treatment is based on results of CRP (*C-reactive protein)* - **PCT** = in case the treatment is based on results of PCT (*procalcitonin*) - **Other =** in case the treatment is based on results of another lab-based biomarker than CRP, PCT - **WBC =** in case the treatment is based on elevated white blood cell count. Normal number of WBCs in the blood is ± 4,500 to 11,000 WBCs per microliter.   **Type of biological fluid sample:** choose between Blood, Urine or Other.  Complete if available on the day of the PPS also the **most relevant value close to the start of the antibiotic treatment** (numeric optional field) in mg/L, μg/L, ng/L, mg/dL, ng/dL, ng/mL, μg/mL, nmol/L. In thousand per microliter (μL) for WBC count.  For conversion calculator see: <http://unitslab.com/node/67> (CRP) and <http://unitslab.com/node/103> (procalcitonin).  **Culture(s) sent to the lab to document infection** – Specify the origin of the sample. *Optional field.*  8 ***multiple choice*** answers are possible:   - Blood culture - Cerebrospinal fluid (CSF) - Urine : sterile midstream and catheter specimen sample - Wound (surgery/biopsy): sample obtained via surgery, incision, soft tissue biopsy, closed abscess, **no** swabs - Broncho-alveolar lavage (BAL) – protected respiratory specimen - Sputum or bronchial aspirate - Stool - Other type of specimen   ***Data collected at antimicrobial level***  **Antimicrobial Drug Name** – This is the generic name (e.g. amoxicillin and beta-lactamase inhibitor and not Augmentin^®^). Antimicrobials for ***topical use*** applied on the skin/eye/ear etc are **not** included. The antimicrobial data are automatically recorded in the Global-PPS tool with its ATC5 code following the ATC classification system of the WHO Collaborating Centre for Drug Statistics. (<https://www.whocc.no/atc_ddd_index/>).  **Antimicrobials included in the survey are** (see also antimicrobial list available at <https://www.global-pps.com/documents/>):   1. Antibacterials for systemic use: J01 2. Antimycotics and antifungals for systemic use: J02 and D01BA (including griseovulvine and terbinafine) 3. Drugs for treatment of tuberculosis: J04A (these are the antibiotics as well as all other drugs to treat tuberculosis) 4. Antibiotics used as intestinal anti-infectives: A07AA 5. Antiprotozoals used as antibacterial agents, nitroimidazole derivatives: P01AB 6. Antivirals for systemic use: J05 7. Antimalarials: P01B   **Start date of the antimicrobial** : *dd/mm/yyyy – Optional field*  **Administered “Single Unit Dose”** **and “Unit” of Dose** – Administered dose is the actual prescribed single unit dose per administration, expressed in mg, g, IU or MU. Provide number of times/day given in next variable.   - For **combination with one active ingredient as the main antimicrobial agent**, like penicillins with beta-lactamase inhibitors, only the content of active ingredient should be recorded and entered in the Global-PPS tool. E.g. amoxicillin and beta-lactamase inhibitor 500/125 (amoxicillin 500 mg and clavulanic acid 125 mg as potassium salt) should be entered as 500 mg. Important: this must still be listed as amoxicillin and beta-lactamase inhibitor and NOT amoxicillin! - For **combinations with two or more active ingredients** like sulfamethoxazole and trimethoprim, the total content should be entered in *Global-PPS tool*. For example sulfamethoxazole 200 mg/ trimethoprim 40 mg will be recorded as 240 mg.   **Combinations of an antibiotic and an enzyme inhibitor:**  J01CR01 Ampicillin and beta-lactamase inhibitor: report only ampicillin dose  J01CR02 Amoxicillin and beta-lactamase inhibitor: report only amoxicillin dose  J01CR03 Ticarcillin and beta-lactamase inhibitor: report only ticarcillin dose  J01CR05 Piperacillin and beta-lactamase inhibitor: report only piperacillin dose  Examples can be found on: <https://www.whocc.no/ddd/list_of_ddds_combined_products/>  **Times per Day** – This refers to the number of actual prescribed doses per 24 hours. For example every 6 hours = 4; every 8h = 3, every 12h = 2, every 16h = 1.5, every 36h = 0.67, and every 48h = 0.5 doses per day.  **Route** – Route of Administration. Five routes of administration are included: Intravenous and intrathecal and intraperitoneal=P, Intramuscular=IM, Oral=O, Rectal=R, Inhalation=I. For analyses intravenous, intrathecal and intramuscular are all parenteral use (=P).  **Diagnosis** – This is the reason to treat the patient (See appendix II, page 7 of data collection templates). Select ONLY ONE of the possibilities. If more categories are possible, write the one most applicable. Request additional information from doctors, nurses or pharmacists if needed.  **Type of indication** – Refers to whether it concerns therapeutic treatment (Community Acquired Infection=CAI or Healthcare-Associated Infection=HAI) or prophylactic use (Medical or surgical). The indication should be found in the records and/or obtained from ward staff (See appendix III, page 8 of data collection templates for all available codes).   - For **surgical patients**, administration of antimicrobial prophylaxis should be checked in the previous 24 hours in order to encode the duration of prophylaxis as either one dose, one day (=multiple doses given over 24 hours) or >1 day (Important: see protocol p 7-8, inclusion criteria for surgical patients)!   **Reason in notes –** “Yes” or “No”. It refers to whether or not a diagnosis or indication for treatment or prophylaxis was recorded in the medical records when the antimicrobial treatment started; and is based on the information available in the notes. **It should be completed without asking anyone, and derived from records only.**  **Missed Doses -** *Optional field* - Number of missed doses from the start date of the current antibiotic treatment until the date of the survey. If no doses missed, report as 0. If unknown, leave field empty.  **Reason for missed doses -** *Optional field -* due to stock out (S), the patient could not purchase (P), the patient declined/refused the antimicrobial (D), other reason (O), multiple (mixed) reasons (M), unknown (U)  ***Example***: The antibiotic treatment started 2 days before the survey, one tablet 3 times daily. On the second day, the second and third tablets were not administered due to unavailability. Missed doses = 2 and Reason = S (stock out issues).  **Guideline compliance** – This depends on whether **the antibiotic choice** is in compliance with local guidelines Y=Yes (compliant with local policy or infection specialist advice); N=No; NA=Not Assessable (no local guidelines for the specific indication); NI=No Information (because the indication is unknown). Thus, appropriateness refers EXCLUSIVELY to drug choice, not the dose, route, or duration, as this is more contentious.   - Note: Therapy directed by an infection specialist is “Yes-compliant”. - Note: If the choice of drug agrees only partially with guideline the entire combination therapy or prophylaxis should be classified as non-compliant.   **Is a stop/review date documented?** “Yes” or “No”. It concerns whether a date of review or stop date of the antimicrobial was recorded in the medical records. This has to be filled-in for all included antimicrobials even if it is long term medical prophylaxis where a stop/review day is unlikely.  **Type of treatment** – E versus T : Note down the information which is “available” at the time of survey. The field is optional for medical prophylaxis (MP) or surgical prophylaxis (SP).   - Empirical treatment (E)   - when the antibiotic is being used as per a local guideline – as a best guess - treatment by means which experience has proved to be beneficial   - when a culture or microbiological examination is done, but the result is not yet available on the day of the PPS (e.g. confirmation of positive blood culture arrives a day after the survey); or the result was not assessable (no growth) - Targeted treatment (T)   - based upon microbiological result. Microbiology result can be any culture and/or sensitivity result from a relevant clinical (e.g., blood, sputum, etc.,) [BUT not screening] specimen as well as any other microbiology result like for example Legionella Urinary Antigen.   - Report also Targeted if the micro-organism yielded susceptible results.   - Report also Targeted if antibiogram is seen (before 8am day of the PPS), but no adaptation of the antibiotic treatment (e.g. according to the sensitivity result de-escalation was possible).   **When treatment choice is based on microbiological data (treatment = targeted),** complete the detected micro-organisms as well as the resistance type concerned. Maximum 3 micro-organisms; and for each of them 1 resistance type can be recorded.  Report the micro-organism, if available on the day of the PPS, also if the pathogens tested were sensitive!  The list of micro-organisms by resistance type are provided in Appendix IV, page 9 of the data collection templates.  Twelve resistance types can be reported:   - **Targeted treatment against MRSA** –It refers to whether the chosen drug is targeting methicillin-resistant *Staphylococcus aureus*. - **Targeted treatment against MRCoNS** – It refers to whether the chosen drug is targeting methicillin-resistant coagulase-negative staphylococci. - **Targeted treatment against PNSP** – It refers to whether the chosen drug is targeting Penicillin-nonsusceptible *Streptococcus pneumoniae*. - **Targeted treatment against MLS** – It refers to whether the chosen drug is targeting macrolide-lincosamide-streptogramin resistance in Streptococcus isolates. - **Targeted treatment against VRE** – It refers to whether the chosen drug is targeting vancomycin-resistant enterococci. - **Targeted treatment against ESBL-producing Enterobacterales** – ‘Yes’ versus ‘No’. It refers to whether or not the chosen drug is targeting Enterobacterales producing extended-spectrum beta-lactamase. - **Targeted treatment against 3^rd^ generation cephalosporin-resistant Enterobacterales.** It refers to whether the chosen drug is targeting 3^rd^ generation cephalosporin-resistant Enterobacterales. - **Targeted treatment against Carbapenem-resistant Enterobacterales** - It refers to whether the chosen drug is targeting Carbapenem-resistant Enterobacterales. - **Targeted treatment against ESBL-producing nonfermenter Gram-negative bacilli** – It refers to whether the chosen drug is targeting nonfermenters (*Pseudomonas aeruginosa, Acinetobacter baumannii, Burkholderia spp., Stenotrophomonas maltophilia*) producing extended-spectrum beta-lactamase. - **Targeted treatment against carbapenem-resistant non fermenter Gram-negative bacilli** – It refers to whether the chosen drug is targeting carbapenem-resistant nonfermenters (*Pseudomonas aeruginosa, Acinetobacter baumannii, Burkholderia spp., Stenotrophomonas maltophilia*). - **Targeted treatment against other MDR organisms** – It refers to whether the chosen drug is targeting multidrug-resistant (MDR) organisms, other than the ones listed above. An organism can be considered as MDR if it is non-susceptible to at least 3 antimicrobial classes that are usually considered as suitable treatment options for that pathogen. For tuberculosis, consider as MDR if it does not respond to at least isoniazid and rifampicin.^[[6]](#footnote-6),^^[[7]](#footnote-7)^ - **Targeted against azole-resistant fungi/yeasts** – It refers to whether the chosen drug is targeting **azole** drug resistance.  The HAI-PATIENT form: Optional HAI module Supplementary information can be collected **for each patient receiving at least one antimicrobial** to allow investigation of Healthcare-Associated Infections (HAI) in more detail with a specific focus on invasive devices.  The following variables need to be completed:  **Date of admission in the hospital** (optional)*: dd/mm/yyyy*  **Previous hospitalization < 3 months** (optional): Yes-ICU, Yes-other, No, Unknown  **Surgical procedure during current admission in hospital** (mandatory): Yes, No, Unknown  Surgery is defined as the treatment of injuries or diseases by cutting open the body and removing or repairing the damaged part (American dictionary). Diagnostic interventions as such are not scored.  **Previous antibiotic course < 1 month** (optional): Yes, No, Unknown  **Presence of an invasive** device **at 8 am** on the day of the PPS.  Definition of **“*an (inserted) invasive device*”**:   - - **Report also** a patient with a device with intermittent insertion, and “accidentally or intentionally” removed before 8am but planned re-insertion after 8am.   - **Do not report** a patient with a device with planned (intentional) removal before 8am and whereby no re-insertion is planned after 8am.   **Six invasive devices are surveyed** (Mandatory)**:**   - - Indwelling Urinary Catheter: includes urethral and suprapubic catheters as well as intermittent catheters. Exclude external catheters which do not enter the urethra such as condom catheter   - Peripheral Vascular / Intravenous Catheter (at least one)   - Central Vascular Catheter^[[8]](#footnote-8)^. Exclude implantable venous access port   - Invasive respiratory endotracheal intubation. Include tracheostomy   - Non-invasive positive and negative mechanical ventilation (CPAP, BiPAP, CNEP, ….)   - Inserted tubes and drains (T/D): include nephrostomy tubes, intra-abdominal tubes and drains, cerebrospinal fluid shunts. Exclude feeding tubes.   For each device, indicate whether present for the patient (Yes, No, Unknown) and optionally complete the date of 1^st^ insertion/start date of the device.  **McCabe Score** (mandatory)^[[9]](#footnote-9)^: Classifies the severity of underlying medical conditions. Disregard the influence of acute infections, e.g. if the patient has an active infection, estimate the score the patient had before the infection. ***Choose between the categories***: Non-fatal disease (expected survival at least five years); ultimately fatal disease (expected survival between one and five years); rapidly fatal disease (expected death within one year) or unknown.  **Underlying morbidity** (multiple choice, mandatory); refers mainly to immunocompromised patients. List of underlying morbidities is provided in the data collection templates, page 4. HOSPITAL PROFILE: Optional data to collect at hospital level Institutional characteristics influence antimicrobial prevalence and the prevalence of HAI. With this optional module we encourage hospitals to collect and evaluate a number of structure and process indicators at the hospital level. The collected data will allow them to examine available workforce, equipment and various support available at hospital level which might facilitate antimicrobial stewardship interventions as well as patient safety.  See data collection forms, Page 5, for an overview of indicators. Information which is not available can be left open; all indicators can optionally be completed. These data can be entered online on a yearly basis. **EXPORT YOUR DATA** Data can be exported to a Microsoft Excel^®^ file. One can do this at any time during the process of data entry. It contains the raw recorded department (denominator) and patient (numerator) data. It allows the user/s to verify own data (correctness and completeness of data). It also enables hospitals to perform analysis on own data.  Data are reported in 3 excel worksheets:   - Institution: provides details on the institution - Departments: provides details on denominators collected at ward level. - Patients: provides details at antimicrobial level for each patient receiving at least one antimicrobial on the day of the survey. Attention, each line is one antimicrobial. The information collected at patient level such as age, sex are repeated on the corresponding lines for a certain patient. A unique patient is defined by their survey number.  **VALIDATION PROCESS** After the denominator data and all patient data have been entered to the Global-PPS tool, the participant needs to complete the validation process in order to be able to generate a feedback report. The validation process identifies small, basic issues, warnings or errors in the survey.  For example:   - Surveys without data entry gives an error - Incomplete departments (missing values, denominator values) gives an error - Patient data without antibiotic treatments or with duplicated antibiotics gives an error - Patient data without HAI forms completed (only if one participates in the HAI module) gives an error - Warnings about extremely high dosing values. - Check on consistency between the diagnostic code and the indication (therapeutic versus prophylactic use): gives a warning - Check on Targeted prescribing for surgical prophylaxis and medical prophylaxis: gives a warning  **FEEDBACK** The Global-PPS tool is designed to produce an automated feedback for each participating hospital. We anticipate sending data back in a simple, easy-to-use feedback, with own data ready to use for local presentations. The feedback compares hospital data to i] National (if N≥3 institutions) and ii] Continental results. Hospitals participating a second time or more are able to download a longitudinal feedback report. Participants (local administrators) entering data for more than one hospital are able to download merged feedback reports.  Feedback reports can only be produced if the hospital data have been validated. The feedback report includes various tables and charts on the prevalence of antimicrobial use, resistance and healthcare-associated infections; as well as quality indicators of appropriate antimicrobial use. An example is available at <https://www.global-pps.com/documents/> |
| --- | --- | --- | --- | --- | --- | --- |

## **Appendix II –** Data collection forms of the basic inpatient module and healthcare-associated infection (HAI) module of the Global Point Prevalence Survey (Global-PPS)

| **Ward Form** (Mandatory: Fill in one form for each ward included in the PPS)  **Include only inpatients “admitted before and present at 08:00 hours” on the day of the PPS!**   \| **Date of survey** (dd/mm/year) \| \| _____/_____/________ \| **Person completing form** (Auditor code) : \| \| \|  \| \| \| --- \| --- \| --- \| --- \| --- \| --- \| --- \| --- \| \| **Hospital name :** \| \|  \| **Ward Name :** \| \| \|  \| \| \| **Ward Type:**  Tick the most appropriate type of department / ward \| **Adult wards** \| \| \| \| \| **Paediatric wards** \| \| \| **AMW** (General or mixed **Adult Medical Ward**)  **HO-AMW** (Haematology-Oncology)  **T-AMW** (Transplant (BMT/solid))  **P-AMW** (Pneumology)  **CAR-AMW** (Cardiology)  **NEU-AMW**(Neurology)  **REN-AMW** (Nephrology)  **ID-AMW** (Infectious Disease)  **DB-AMW** (Dermatology-burn wards)  **PSY-AMW** (Psychiatry)  **REH-AMW** (Rehabilitation)  **GER-AMW** (Geriatrics)  **LTC-AMW** (Long-Term care)  **OBG-AMW** (gynaecology-obstetrics)  **IS-AMW** (Isolation ward, e.g. COVID patients)  **ASW** (General or mixed **Adult Surgical Ward**)  **DIG-ASW** (Digestive tract surgery)  **ORT-ASW** (Orthopaedics-Trauma surg.)  **URO-ASW** (Urological surg.)  **CV-ASW** (Cardio-vascular surg.)  **NEU-ASW** (Neurosurgery)  **ONCO-ASW** (Oncology-cancer surg.)  **PLAS-ASW** (Plastic, reconstructive surg.)  **ENT-ASW** (Ear-nose-throat surg.)  **AICU** (General or mixed **Adult Intensive Care Unit**)  **MED-AICU** (Medical AICU)  **SUR-AICU** (Surgical AICU)  **CAR-AICU** (Cardiac AICU)  **AHDU** (High Dependency Unit) \| \| \| \| \| **PMW** (Paediatric Medical Ward)  **HO-PMW** (Haematology-Oncology)  **T-PMW** (Transplant (BMT/Solid))  **PSW** (Paediatric Surgical Ward)  **PICU** (Paediatric Intensive Care Unit)  **ID-PMW** (Infectious Disease PMW)  **Neonatal wards:**  **NMW** (Neonatal Medical Ward)  **NICU** (Neonatal Intensive Care Unit) \| \| \| **Mixed ward** \| **Yes**  **No** \| \| \| \| \| \| \| \| **Activity**: Tick as appropriate. In case of mixed wards, tick all encountered activities/specialties \| \| \| \| **Medicine** \| **Surgery** \| \| **Intensive Care** \| \| **Total number of admitted inpatients** **(=all patients whether they receive an antimicrobial or not!)** on the ward present at 8.00 am on day of PPS. For mixed departments, fill the total number of patients corresponding to each of the encountered activities. \| \| \| \|  \|  \| \|  \| \| **Total number of beds** on the ward present at 8:00 am on day of PPS split up by activity. For mixed departments fill in the total number of beds corresponding to each of the encountered activities. \| \| \| \|  \|  \| \|  \|   **GLOBAL-PPS PATIENT Form**  (Mandatory: Fill in one form per patient with an active/ongoing antimicrobial at 8am on the day of the PPS)   \| **Ward** **Name/code** \| **Activity** ^1^  (M, S, IC) \| **Patient Identifier** ^2^ \| **Survey Number** ^3^ \| \| **Patient Age** ^4^ \| \| \| \| --- \| --- \| --- \| --- \| --- \| --- \| --- \| --- \| \| **Years**  ≥ 2 years \| **Months**  1-23 month \| **Days**  <1 month \| \|  \|  \|  \|  \| \|  \|  \|  \| \| ***Current Weight**** *In kg* \| \| ***Neonate only*** *(optional)* \| \| \| \| **Sex** (M, F, U) \| \| \| ***Gestational age**** \| \| ***Birth weight**** *(kg)* \| \| \|  \|  \|  \|  \| \|  \|  \|  \|  \| **Treatment based on biomarker data or WBC** \| \| \| 0 Yes 0 No \| \| **Culture(s) sent to the lab to document infection* (Tick if yes)** \| \| \| --- \| --- \| --- \| --- \| --- \| --- \| --- \| \| **If yes, which:**  CRP, PCT, other, WBC^5^ \|  \| **Most relevant value close to start antimicrobial**  **Value Unit^6^** \| \| \| \| **Blood**  **Urine**  **Stool**  **Cerebro-spinal fluid** \| **Wound** (surgery/biopsy)  **BAL** (protected resp. specimen)  **Sputum/bronchial aspirate**  **Other type of specimen** \| \| **Type biological fluid sample** (Blood/urine/  other) \|  \|  \| \|  \|  \| **Antimicrobial Name** ^7^ \| **1.** \| **2.** \| **3.** \| **4.** \| **5.** \| \| --- \| --- \| --- \| --- \| --- \| --- \| \| **Start date of the antimicrobial*** (*dd/mm/yyyy)* \|  \|  \|  \|  \|  \| \| **Single Unit Dose** ^8^ \|  \|  \|  \|  \|  \| \| **Unit** (g, mg, IU, MU) ^9^ \|  \|  \|  \|  \|  \| \| **Doses/ day** ^10^ \|  \|  \|  \|  \|  \| \| **Route** (P, O, R, I, IM)^11^ \|  \|  \|  \|  \|  \| \| **Diagnosis** ^12^ (see appendix II) \|  \|  \|  \|  \|  \| \| **Type of indication** ^13^ (see appendix III) \|  \|  \|  \|  \|  \| \| **Reason in Notes** (Yes or No) ^14^ \|  \|  \|  \|  \|  \| \| **Guideline Compliance** (Y, N, NA, NI) ^15^ \|  \|  \|  \|  \|  \| \| **Is a stop/review date documented?** (Yes/No) \|  \|  \|  \|  \|  \| \| **N missed doses***^16^ \|  \|  \|  \|  \|  \| \| **Reason* (**S,P,D,O,M,U)^17^ \|  \|  \|  \|  \|  \| \| **Treatment** (E: Empirical; T: Targeted)^18^ \|  \|  \|  \|  \|  \|     **The following resistance data is to be filled in only if the treatment choice is based on microbiology data**  **(Treatment=T) available on the day of the PPS**  Maximum 3 microorganisms (MO) to report Maximum 1 Resistance type by MO to report   \| **Insert codes (**Appendix IV) \|  \| **MO** \| **R type^**^** \| **MO** \| **R type^**^** \| **MO** \| **R type^**^** \| **MO** \| **R type^**^** \| **MO** \| **R type^**^** \| \| --- \| --- \| --- \| --- \| --- \| --- \| --- \| --- \| --- \| --- \| --- \| --- \| \| **MO1** \|  \|  \|  \|  \|  \|  \|  \|  \|  \|  \| \| **MO2** \|  \|  \|  \|  \|  \|  \|  \|  \|  \|  \| \| **MO3** \|  \|  \|  \|  \|  \|  \|  \|  \|  \|  \| \| ***Resistance type^**^***- choose between: **MRSA**^19^; **MRCoNS**^20^**; PNSP**^21^**; MLS**^22^**; VRE**^23^; **ESBL** (ESBL-producing Enterobacterales^24^)**; 3GCREB** (3^rd^ generation cephalosporin resistant Enterobacterales); **CRE** (Carbapenem-resistant Enterobacterales^25^)**; ESBL-NF** (ESBL-producing non fermenter Gram-negative bacilli^26^); **CR-NF** (Carbapenem-resistant non fermenter Gram-negative bacilli ^27^); **other MDRO**^28^; **Azoles**^29^. Encode Microorganism also if resistance type is unknown.  **Note: *** *Current weight*, Gestational age (in number of weeks), *Birth weight*, *Start date of the antimicrobial* and *Cultures sent to the lab, missed doses* are **optional variables**. \| \| \| \| \| \| \| \| \| \| \| \|   1 Activity: M=medicine (including Psychiatric cases, *etc.*), S=surgery (including orthopaedics, obstetrics and gynaecology, *etc.*), IC=intensive care  2 Patient Identifier: A unique patient identifier that allows linkage to patient records at local level for more detailed audit. This unique identifier will not be included in the online database.  3 Survey Number: A unique non-identifiable number given by WebPPS for each patient entered in the database. Leave blank but note down the number after the patient data has been recorded in the online database. The number is displayed once (and only) after the patient data has been recorded in the online database.  4 Patient Age: If the patient is 2 years old or older, specify only the number of years, if between 1 and 23 months specify only the number of months, if less than 1 month specify the number of days.  ^5^  If treatment based on biomarker, specify which one: **CRP** (C-reactive protein), **PCT** (Procalcitonin), **Other** lab-based biomarker other than CRP, PCT; or **WBC** (white blood cell count).  ^6^ The unit for the biomarker CRP or PCT value expressed in mg/L, μg/L, ng/L, mg/dL, ng/dL, ng/mL, μg/mL, nmol/L. In thousand per microliter (μL) for WBC count (normal number of WBCs in the blood is 4,500 to 11,000 WBCs per microliter). For a conversion calculator see: <http://unitslab.com/node/67> (CRP) and <http://unitslab.com/node/103> (procalcitonin).  ^7^ Antimicrobial Name: Insert generic name.  8 Single Unit Dose: Numeric value for dose per administration (in grams, milligrams, IU or MU).  ^9^  Unit: The unit for the dose (g, mg, IU or MU)  ^10^  Doses/day^:^ If necessary provide fractions of doses: (e.g., every 16h = 1.5 doses per day, every 36h = 0.67 doses per day, every 48h = 0.5 doses per day)  ^11^ Route: Routes of administration are: Intravenous and intrathecal and intraperitoneal=P, Intramuscular=IM, Oral=O, Rectal=R, Inhalation=I. See also protocol page 18  ^12^ See diagnoses groups list (Appendix II)  ^13^ See Indication codes (Appendix III)  ^14^ Reason in Notes**:** A diagnosis / indication for treatment is recorded in the patient’s documentation (treatment chart, notes, etc.) at the start of antibiotic course (Yes or No)  ^15^  Guideline Compliance: Refers to antibiotic choice (not route, dose, duration etc) in compliance with **local** guidelines (Y: Yes; N: No; NA: Not Assessable because of absence of local guidelines for the specific indication; NI: No Information because diagnosis/indication is unknown)  ^16^ N missed doses: Number of missed doses from start date of current antibiotic treatment until the date of the survey. If no doses missed, report as 0. If unknown, leave field empty.  ^17^ Reason: Reason for missed doses: due to **stock** out (S), patient could not **purchase** (P), patient **declined**/refused (D), **other** reason (O), **multiple** reasons (M), **unknown** (U).  ^18^ Treatment: **Report “E”** 1) when the antibiotic is being used as per a local guideline, treatment by which experience has proved to be beneficial; 2) when a culture or microbiological examination is not done ; 3) when a microbiological examination is done, BUT not yet available on the day of the PPS; or the result was not assessable. **Report “T”** if based upon microbiological result; Report also “T” if the micro-organism yielded susceptible results.  ^19^ Methicillin-resistant *Staphylococcus aureus*  (MRSA)  ^20^  Methicillin-resistant coagulase negative staphylococci (MRCoNS)  ^21^ Penicillin-non susceptible *Streptococcus pneumoniae* (PNSP)  ^22^ Macrolide-lincosamide-streptogramin resistance in Streptococcus isolates (MLS)  ^23^  Vancomycin-resistant enterococci (VRE)  ^24^ Bacteria, producing extended-spectrum beta-lactamases (ESBL)  ^25^ Carbapenem-resistant *Enterobacterales* (CRE) – enteric bacteria resistant to imipenem, meropenem or other carbapenems  ^26^ ESBL Non fermenters (ESBL-NF): *Pseudomonas aeruginosa, Acinetobacter baumannii, Burkholderia spp., Stenotrophomonas maltophilia* multidrug resistant  ^27^ Carbapenem-resistant Nonfermenters (CR-NF) – nonfermenters resistant to imipenem, meropenem or other carbapenems  ^28^ Multi-drug resistant (MDR) pathogens, others than the listed above  ^29^ Azoles: if the medicinal product chosen is intended to treat infections caused by azole-resistant fungi and yeasts (e.g*. Candida spp*., *Aspergillus spp*.)  **GLOBAL-PPS PATIENT Form – additional variables for HAI at patient level**  (Mandatory: Fill in one form per patient with an active/ongoing antimicrobial at 8am on the day of the PPS)   \| **Ward** **Name/code** \| **Activity** ^1^  (M, S, IC) \| **Patient Identifier** ^2^ \| **Survey Number** ^3^ \| \| **Patient Age** ^4^ \| \| \| \| --- \| --- \| --- \| --- \| --- \| --- \| --- \| --- \| \| **Years**  ≥ 2 years \| **Months**  1-23 month \| **Days**  <1 month \| \|  \|  \|  \|  \| \|  \|  \|  \| \| ***Current Weight**** *In kg* \| \| ***Neonate only*** *(optional)* \| \| \| \| **Sex** (M, F, U) \| \| \| ***Gestational age**** \| \| ***Birth weight**** *(kg)* \| \| \|  \|  \|  \|  \| \|  \|  \|  \|  \| **Date of admission in the hospital** (dd/mm/yyyy) (optional) \| \|  \| \| \| \| \| --- \| --- \| --- \| --- \| --- \| --- \| \| **Previous hospitalization < 3 months** (optional) \| Yes, ICU \| \| Yes, Other \| No \| Unknown \| \| **Surgical procedure during current admission in hospital** \| \| \| Yes \| No \| Unknown \| \| **Previous antibiotic course < 1 month** (optional) \| \| \| Yes \| No \| Unknown \|  \| **“Inserted” invasive device present at 8 am on the day of the PPS** \| \| \| \| **Date 1^st^ insertion** \| \| --- \| --- \| --- \| --- \| --- \| \| Indwelling Urinary Catheter (UC) \| Yes \| No \| Unknown \| *__/___/____* \| \| Peripheral Vascular / intravenous Catheter (PVC) \| Yes \| No \| Unknown \| *__/___/____* \| \| Central Vascular Catheter (CVC) \| Yes \| No \| Unknown \| *__/___/____* \| \| Non-invasive pos. & neg. mechanical ventilation (CPAP, BiPAP, CNEP, …) \| Yes \| No \| Unknown \| *__/___/____* \| \| Invasive respiratory endotracheal intubation (IRI) \| Yes \| No \| Unknown \| *__/___/____* \| \| Inserted tubes and drains (T/D) \| Yes \| No \| Unknown \| *__/___/____* \| \| Indwelling Urinary Catheter (UC) \| Yes \| No \| Unknown \| *__/___/____* \| \| Peripheral Vascular / intravenous Catheter (PVC) \| Yes \| No \| Unknown \| *__/___/____* \|  \| **McCabe score** \| Non-fatal disease \| Ultimately fatal disease \| Rapidly fatal disease \| UNK/Not available \| \| --- \| --- \| --- \| --- \| --- \|  \| **Underlying morbidity** *(multiple choice, maximum 3 choices)* \| \| \| --- \| --- \| \| Diabetes mellitus, type 1 or 2 \| High dose steroids^^[[10]](#footnote-10)^^ \| \| AIDS/HIV (only if last CD4 count <500/mm^3^) \| Malnutrition^^[[11]](#footnote-11)^^ \| \| Hematological or solid cancer/ Recent chemotherapy (<3months) \| Long COVID \| \| End-stage Liver Disease, cirrhosis \| \| Stem cell or solid organ transplant \| Trauma \| \| Chronic Renal Disease (all stages) \| Gastroenterological disease (inflammatory bowel disorders, Coeliac disease,…) \| \| Active tuberculosis \| \| Genetic disorder \| Chronic neurological conditions^^[[12]](#footnote-12)^^ \| \| Congenital heart diseases \| Other \| \| Chronic lung diseases including cystic fibrosis, COPD, bronchiectasis, asthma \| None \| \| Neutropenia \| Unknown \|   **HOSPITAL PROFILE – “Optional data” to be collected at hospital level**  **Provide, if available, for each indicator the year of reference and the number “at hospital level”.**   \|  \| **Year** (yyyy) \| **Number** \| \| --- \| --- \| --- \| \| Hospital size: number (N) beds \|  \|  \| \| Number of admissions (or discharges)/year \|  \|  \| \| Number of patient days or occupied bed-days/year \|  \|  \| \| Number of consumption of alcohol-based hand rub in litres/year \|  \|  \| \| Number of “patients” with blood culture test/year \|  \|  \| \| Number of stool tests for *Clostridioides difficile* Infections/year \|  \|  \| \| Number of FTE* antimicrobial stewardship physicians \|  \|  \| \| Number of FTE antimicrobial stewardship pharmacists \|  \|  \| \| Number of FTE Infection prevention control (IPC) doctors \|  \|  \| \| Number of FTE Infection prevention control (IPC) nurses \|  \|  \|   *FTE=Full-Time Equivalent units or equivalent employees working full-time on antimicrobial stewardship activities or IPC. E.g. if 3 employees work 20 hours, 30 hours and 10 hours/week=total 60 hours/week and assuming that a full-time employee works 40hours/week, the FTE calculation equals 60hours/40hours; or 1.5 FTE  **Indicate for each indicator at hospital level if available ‘yes’ or ‘no’.**   \|  \| **Yes** \| **If yes: Year of introduction** \| **No** \| \| --- \| --- \| --- \| --- \| \| Presence of formally defined AMS* program \|  \|  \|  \| \| Presence of active AMS group (committee and operational team) \|  \|  \|  \| \| Presence of formally defined IPC* program \|  \|  \|  \| \| Presence of active IPC group (committee and operational team) \|  \|  \|  \| \| Presence of regular IPC (annual, quarterly) feedback to health care workers \|  \|  \|  \| \| Clinical Infectious Disease (ID) consultation available \|  \|  \|  \| \| Specialized AMS or ID training available for physicians/pharmacists \|  \|  \|  \| \| Presence of microbiology lab support on site \|  \|  \|  \| \| Availability of microbiology lab on weekends/holidays \|  \|  \|  \| \| Availability of periodic cumulative antimicrobial susceptibility report** \|  \|  \|  \| \| If yes, is susceptibility report distributed to prescribers? \|  \|  \|  \| \| Availability of standardized criteria for appropriate IV-PO switch \|  \|  \|  \| \| Software available for Infection Control and/or AMS \|  \|  \|  \| \| Presence of bundles or checklists to decrease CAUTI, VAP, CR-BSI, CDIF, SSI° \|  \|  \|  \|   *AMS=Antimicrobial Stewardship; IPC=Infection Prevention and Control; ** local epidemiological report;  ° CAUTI=Catheter Associated Urinary Tract Infection; VAP=Ventilator Associated Pneumonia; CR-BSI=Catheter-related Blood Stream Infection; CDIF= Clostridioides Difficile Infection; SSI=Surgical Site Infections.  **Tick for each indicator if available at hospital level.**   \| Availability of **written policy to document the antimicrobial prescription** in the medical record \| - Yes, all wards \| - Yes, selected wards \| - Yes, in ICU \| - No \| \| --- \| --- \| --- \| --- \| --- \| \| Availability of **formal restriction procedure** (defined formulary, restrictive list) for certain antimicrobials \| - Yes, all wards \| - Yes, selected wards \| - Yes, in ICU \| - No \| \| Presence formal review of antimicrobial **after 48 hours** (**post-prescription review**) \| - Yes, all wards \| - Yes, selected wards \| - Yes, in ICU \| - No \| \| Presence of **antimicrobial ward rounds** (Review of antimicrobial orders for assigned patients) \| - Yes, all wards \| - Yes, selected wards \| - Yes, in ICU \| - No \| \| Who can prescribe antibiotics in your hospital? \| - Physician \| - Pharmacist \| - Nurse \| - Other \|   **Appendix I: Combination anti-infective agents**  **Combinations of an antibiotic and a beta-lactamase inhibitor:**   - Do not report the dose of the beta-lactamase inhibitor   Ampicillin and beta-lactamase inhibitor: **report only ampicillin dose** (J01CR01)  Amoxicillin and beta-lactamase inhibitor: **report only amoxicillin dose** (J01CR02)  Ticarcillin and beta-lactamase inhibitor: **report only ticarcillin dose** (J01CR03)  Piperacillin and beta-lactamase inhibitor: **report only piperacillin dose** (J01CR05)  Imipenem and beta-lactamase inhibitor: **report only imipenem dose** (J01DH51)  Panipenem and betamipron: **report only panipenem** (J01DH55)  Example:   - Amoxicillin and beta-lactamase inhibitor 1.2g IV 🡪 1g (amoxicillin) + 200mg (clavulanic acid), **report only 1 g as unit dose** - Piperacillin and beta-lactamase inhibitor 4.5g IV 🡪 4g (piperacillin) + 500mg (tazobactam), **report only 4 g as unit dose**   **Other combinations of multiple antimicrobial substances:**  J01EE01 Sulfamethoxazole and Trimethoprim: **report the total amount of sulfamethoxazole and trimethoprim**  Example:   - Co-trimoxazole 960mg: (sulfamethoxazole. 800mg + trimethoprim 160mg), **report 960mg**   Further information on agents included for the Global-PPS is available in the antimicrobial list. Only antimicrobial substance name needs to be written down, NOT the ATC codes! (excel file - available on website under documents: Global-PPS_antimicrobial_list.xlsx) <http://www.global-pps.com/>  **Appendix II -** **Diagnostic therapeutic / treatment codes (what the clinician aims at treating)**   \| **Site** \| **Codes** \| **Examples** \| \| --- \| --- \| --- \| \| **CNS** \| **CNS** \| Infections of the **C**entral **N**ervous **S**ystem \| \| **EYE** \| **EYE** \| Therapy for Eye infections e.g., Endophthalmitis \| \| **ENT** \| **ENT** \| Therapy for **E**ar, **N**ose, **T**hroat infections including mouth, sinuses, larynx \| \| **AOM** \| Acute otitis media \| \| **RESP** \| **LUNG** \| Lung abscess including aspergilloma \| \| **URTI** \| **U**pper **R**espiratory **T**ract viral **I**nfections including influenza but not ENT \| \| **Bron** \| Acute **Bron**chitis or exacerbations of chronic bronchitis \| \| **Pneu** \| **Pneu**monia or LRTI (lower respiratory tract infections) \| \| **COVID-19** \| Coronavirus disease caused by SARS-CoV-2 infection \| \| **TB** \| Pulmonary TB (Tuberculosis) \| \| **CF** \| Cystic fibrosis \| \| **CVS** \| **CVS** \| **C**ardio**V**ascular **S**ystem infections: endocarditis, endovascular device e.g pacemaker, vascular graft \| \| **GI** \| **GI** \| Gastro-Intestinal infections (salmonellosis, *Campylobacter*, parasitic, etc.) \| \| **IA** \| **I**ntra­**A**bdominal sepsis including hepatobiliary, intra-abdominal abscess *etc*. \| \| **CDIF** \| *Clostridioides difficile* infection \| \| **SSTBJ** \| **SST** \| **S**kin and **S**oft Tissue: Cellulitis, wound including surgical site infection, deep soft tissue not involving bone e.g., infected pressure or diabetic ulcer, abscess \| \| **BJ** \| **B**one/**J**oint Infections: Septic arthritis (including prosthetic joint), osteomyelitis \| \| **UTI** \| **Cys** \| Lower Urinary Tract Infection (UTI) : cystitis \| \| **Pye** \| Upper UTI including catheter related urinary tract infection, pyelonephritis \| \| **ASB** \| Asymptomatic bacteriuria \| \| **GUOB** \| **OBGY** \| **Ob**stetric/**Gy**naecological infections, **S**exually **T**ransmitted **D**iseases (**STD**) in women \| \| **GUM** \| **G**enito-**U**rinary **M**ales + Prostatitis, epididymo­orchitis, STD in men \| \| **Syph** \| Syphilis \| \| **No defined site (NDS)** \| **BAC** \| Bacteraemia or fungaemia with no clear anatomic site and no shock \| \| **SEPSIS** \| Sepsis of any origin (eg urosepsis, pulmonary sepsis etc), sepsis syndrome or septic shock with no clear anatomic site. Include fungaemia (candidemia) with septic symptoms \| \| **Malaria** \|  \| \| **HIV** \| Human immunodeficiency virus \| \| **PUO** \| **P**yrexia of **U**nknown **O**rigin - Fever syndrome with no identified source or site of infection \| \| **PUO-HO** \| Fever syndrome in the non-neutropenic **H**aemato–**O**nco patient with no identified source of pathogen \| \| **FN** \| **F**ever in the **N**eutropenic patient \| \| **LYMPH** \| **Lymph**atics as the primary source of infection eg suppurative lymphadenitis \| \| **Sys-DI** \| Disseminated infection (viral infections such as measles, CMV …) \| \| **Other** \| Antimicrobial prescribed with documentation but no defined diagnosis group \| \| **UNK** \| Completely **Unk**nown Indication \| \| **PROK** \| Antimicrobial (e.g. erythromycin) prescribed for **Prok**inetic use \|   **Appendix II, next - Codes for surgical and medical prophylaxis**   \| **Site** \| **Codes** \| **Examples** \| \| --- \| --- \| --- \| \| **CNS** \| **Proph CNS** \| Prophylaxis for CNS (neurosurgery, meningococcal) \| \| **EYE** \| **Proph EYE** \| Prophylaxis for Eye operations \| \| **ENT** \| **Proph ENT** \| Prophylaxis for **E**ar, **N**ose, **T**hroat (**Surgical or Medical prophylaxis=SP/MP**) \| \| **RESP** \| **Proph RESP** \| Pulmonary surgery, prophylaxis for **Resp**iratory pathogens e.g. for aspergillosis \| \| **CVS** \| **Proph CVS** \| **C**ardiac or **V**ascular Surgery, endocarditis prophylaxis \| \| **GI** \| **Proph GI** \| **G**astro-**I**ntestinal tract surgery, liver/biliary tree, GI prophylaxis in neutropenic patients or hepatic failure \| \| **SSTBJ** \| **Proph BJ** \| Prophylaxis for SST, for plastic or orthopaedic surgery (**B**one or **J**oint) \| \| **UTI** \| **Proph UTI** \| Prophylaxis for urological surgery **(SP)** or recurrent **U**rinary **T**ract **I**nfection **(MP)** \| \| **GUOB** \| **Proph OBGY** \| Prophylaxis for **OB**stetric or **GY**naecological surgery (SP: section caesarean, no episiotomy; MP: carriage of group B streptococcus) \| \| **No defined site (NDS)** \| **MP-GEN** \| Drug is used as **M**edical **P**rophylaxis in **gen**eral, without targeting a specific site, e.g. antifungal prophylaxis during immunosuppression \|   **Appendix II, next - Codes for Neonates**   \| **Site** \| **Codes** \| **Examples** \| \| --- \| --- \| --- \| \| **Neonatal** \| **MP-MAT** \| **M**edical **P**rophylaxis for **Maternal** risk factors e.g. maternal prolonged rupture membranes \| \| **NEO-MP** \| Drug is used as **M**edical **P**rophylaxis for **Newborn** risk factors e.g. VLBW (Very Low Birth Weight) and IUGR (Intrauterine Growth Restriction) \| \| **CLD** \| Chronic lung disease: long-term respiratory problems in premature babies (bronchopulmonary dysplasia) \|   **Appendix III - Type of Indication**   \| **CAI** Community acquired infection  **Device related HAI** \| \| Symptoms started ≤ 48 hours from admission to hospital (or present on admission). \| \| \| \| --- \| --- \| --- \| --- \| --- \| \| **HAI**  Healthcare  Associated  Infection:  Symptoms  start  **48 hours**  **after**  **admission** to hospital \| \| **HAI1** Post-operative surgical site infection (within: 30 days of surgery OR; 90 days after implant surgery) \| \| \| \| **HAI2** ***Intervention*** related infections of mixed origin (mixed infection such as mix of CVC-BSI, PVC-BSI, VAP, CAUTI; or related to tubes/drains) \| \| \| \| **HAI2-CVC-BSI** (Central Venous **Catheter**-related Blood Stream Infection) \| \| \| \| **HAI2-PVC-BSI** (Peripheral Vascular **Catheter**-related Blood Stream Infection) \| \| \| \| **HAI2-VAP** (Ventilator Associated **Pneumonia**) \| \| \| \| **HAI2- CAUTI** (Catheter Associated **Urinary** Tract Infection) \| \| \| \| **HAI3** *C. difficile* associated diarrhoea (CDAD) (>48 h post-admission or <30 days after discharge from previous admission episode. \| \| \| \| **HAI4** Other hospital acquired infection of mixed or undefined origin (HAP, UTI, BSI) \| \| \| \| **HAI4-BSI Blood** Stream Infection, not intervention related \| \| \| \| **HAI4-HAP** Non-intervention related Hospital Acquired **Pneumonia** (not VAP) \| \| \| \| **HAI4-UTI Urinary** Tract Infection, not intervention related \| \| \| \| **HAI5** Patient **referred** from another to the participating hospital with an existing HAI determined and documented on Day 1 of admission or patient **readmitted** <48h after stay in another hospital, with infection present on current admission or within 48 hours (patient with infection from another hospital). \| \| \| \| **HAI6** Infection present on admission from long-term care facility (LTCF) or Nursing Home***** \| \| \| \| **SP** Surgical prophylaxis****** \| \| **SP1** Single dose \| **SP2** one day \| **SP3**  >1 day \| \| For **surgical patients**, administration of prophylactic antimicrobials **should be checked in the previous 24 hours** in order to encode the duration of prophylaxis as either one dose, one day (= multiple doses given within 24 hours) or >1 day.  See more explanation and **table** in **protocol page 8** ! \| \| \| \| \| \| **MP** Medical prophylaxis \| For example long term use to prevent UTI’s or use of antifungals in patients undergoing chemotherapy or penicillin in asplenic patients *etc*. \| \| \| \| \| **OTH** Other \| For example erythromycin as a motility agent (motilin agonist). \| \| \| \| \| **UNK** \| Completely unknown indication \| \| \| \|   **Select 1 possibility for each reported antimicrobial**  *Long-term care facilities represent a heterogeneous group of healthcare facilities, with care ranging from social to medical care. These are places of collective living where care and accommodation is provided as a package by a public-agency, non-profit or private company (e.g. nursing homes, residential homes).  **Surgical prophylaxis includes those antibiotics prescribed before and after a surgical intervention (surgery in the operation room). The code SP1, SP2, SP3 goes with a diagnostic code preceded by ‘proph’ (e.g. ‘proph GI’)  **APPENDIX IV – list of micro-organisms by resistance type**   |
| --- | --- | --- | --- | --- | --- | --- | --- | --- | --- | --- | --- | --- | --- | --- | --- | --- | --- | --- | --- | --- | --- | --- | --- | --- | --- | --- | --- | --- | --- | --- | --- | --- | --- | --- | --- | --- | --- | --- | --- | --- | --- | --- | --- | --- | --- | --- | --- | --- | --- | --- | --- | --- | --- | --- | --- | --- | --- | --- | --- | --- | --- | --- | --- | --- | --- | --- | --- | --- | --- | --- | --- | --- | --- | --- | --- | --- | --- | --- | --- | --- | --- | --- | --- | --- | --- | --- | --- | --- | --- | --- | --- | --- | --- | --- | --- | --- | --- | --- | --- | --- | --- | --- | --- | --- | --- | --- | --- | --- | --- | --- | --- | --- | --- | --- | --- | --- | --- | --- | --- | --- | --- | --- | --- | --- | --- | --- | --- | --- | --- | --- | --- | --- | --- | --- | --- | --- | --- | --- | --- | --- | --- | --- | --- | --- | --- | --- | --- | --- | --- | --- | --- | --- | --- | --- | --- | --- | --- | --- | --- | --- | --- | --- | --- | --- | --- | --- | --- | --- | --- | --- | --- | --- | --- | --- | --- | --- | --- | --- | --- | --- | --- | --- | --- | --- | --- | --- | --- | --- | --- | --- | --- | --- | --- | --- | --- | --- | --- | --- | --- | --- | --- | --- | --- | --- | --- | --- | --- | --- | --- | --- | --- | --- | --- | --- | --- | --- | --- | --- | --- | --- | --- | --- | --- | --- | --- | --- | --- | --- | --- | --- | --- | --- | --- | --- | --- | --- | --- | --- | --- | --- | --- | --- | --- | --- | --- | --- | --- | --- | --- | --- | --- | --- | --- | --- | --- | --- | --- | --- | --- | --- | --- | --- | --- | --- | --- | --- | --- | --- | --- | --- | --- | --- | --- | --- | --- | --- | --- | --- | --- | --- | --- | --- | --- | --- | --- | --- | --- | --- | --- | --- | --- | --- | --- | --- | --- | --- | --- | --- | --- | --- | --- | --- | --- | --- | --- | --- | --- | --- | --- | --- | --- | --- | --- | --- | --- | --- | --- | --- | --- | --- | --- | --- | --- | --- | --- | --- | --- | --- | --- | --- | --- | --- | --- | --- | --- | --- | --- | --- | --- | --- | --- | --- | --- | --- | --- | --- | --- | --- | --- | --- | --- | --- | --- | --- | --- | --- | --- | --- | --- | --- | --- | --- | --- | --- | --- | --- | --- | --- | --- | --- | --- | --- | --- | --- | --- | --- | --- | --- | --- | --- | --- | --- | --- | --- | --- | --- | --- | --- | --- | --- | --- | --- | --- | --- | --- | --- | --- | --- | --- | --- | --- | --- | --- | --- | --- | --- | --- | --- | --- | --- | --- | --- | --- | --- | --- | --- | --- | --- | --- | --- | --- | --- | --- | --- | --- | --- | --- | --- | --- | --- | --- | --- | --- | --- | --- | --- | --- | --- | --- | --- | --- | --- | --- | --- | --- | --- | --- | --- | --- | --- | --- | --- | --- | --- | --- | --- | --- | --- | --- | --- | --- | --- | --- | --- | --- | --- | --- | --- | --- | --- | --- | --- | --- | --- | --- | --- | --- | --- | --- | --- | --- | --- | --- | --- | --- | --- | --- | --- | --- | --- | --- | --- | --- | --- | --- | --- | --- | --- | --- | --- | --- | --- | --- | --- | --- | --- | --- | --- | --- | --- | --- | --- | --- | --- | --- | --- | --- | --- | --- | --- | --- | --- | --- | --- | --- | --- | --- | --- | --- | --- | --- | --- | --- | --- | --- | --- | --- | --- | --- | --- | --- | --- | --- | --- | --- | --- | --- | --- | --- | --- | --- | --- | --- | --- | --- | --- | --- | --- | --- | --- | --- | --- | --- | --- | --- | --- | --- | --- | --- | --- | --- | --- | --- | --- | --- | --- | --- | --- | --- | --- | --- | --- | --- | --- | --- | --- | --- | --- | --- | --- | --- | --- | --- | --- | --- | --- | --- | --- | --- | --- | --- | --- | --- | --- | --- | --- | --- | --- | --- | --- | --- | --- | --- | --- | --- | --- | --- | --- | --- | --- | --- | --- | --- | --- | --- | --- | --- | --- | --- | --- | --- | --- | --- | --- | --- | --- | --- | --- | --- | --- | --- | --- | --- | --- | --- | --- | --- | --- | --- | --- | --- | --- | --- | --- | --- | --- | --- | --- | --- | --- | --- | --- | --- | --- | --- | --- | --- | --- | --- | --- | --- | --- | --- | --- | --- | --- | --- | --- | --- | --- | --- | --- | --- | --- | --- | --- | --- | --- | --- | --- | --- | --- | --- | --- | --- | --- | --- | --- | --- | --- | --- | --- | --- | --- | --- | --- | --- | --- | --- | --- | --- | --- |

## **Appendix III – Calculation of the predictors of the Global-PPS**

**At hospital level:**

- *Type*: hospital type with possible values "Tertiary hospital", "Primary or secondary hospital", "Infectious diseases or specialized hospital";
- *Presence of ICU, transplant or Infectious Diseases Ward:* if one of the following department types is present: AICU, MED-AICU, SUR-AICU, CAR-AICU, AHDU, PICU, NICU, T-AMW, P-AMW, ID-AMW or ID-PMW;
- *Presence of Obstetrics of Gynaecology ward:* if OBG-AMW is present
- *Presence of haematology-oncology ward:* if one of the following department types is present: HO-AMW or HO-PMW;
- *Presence of long-term care ward, geriatrics ward or rehabilitation ward:* if one of the following department types is present: LTC-AMW or GER-AMW, REH-AMW;
- Teaching: if a hospital is a teaching hospital
- *Proportion of admitted adult patients*: number of admitted patients at all AW (= AMW, HO-AMW, T-AMW, P-AMW, CAR-AMW, NEU-AMW, REN-AMW, ID-AMW, DB-AMW, REH-AMW, GER-AMW, LTC-AMW, OBG-AMW, IS-AMW, ASW, DIG-ASW, ORT-ASW, URO-ASW, CV-ASW, NEU-ASW, ONCO-ASW, PLAS-ASW, ENT-ASW, AICU, MED-AICU, SUR-AICU, CAR-AICU, AHDU)/ total number of admitted patients hospital level
- *Proportion of admitted pediatric patients*: number of admitted patients at all PW (=PMW, T-PMW, HO-PMW, PSW, ID-PMW, NMW)/ total number of admitted patients hospital level
- *Proportion of patients admitted at the PICU, NICU or neonatal medical wards*: number of admitted patients at PICU, NICU, NMW/ total number of admitted patients hospital level
- *Total beds*: sum of variable *beds* over all departments within a hospital
- *Total ICU beds*: sum of variable *beds* over the following departments *AICU, MED-AICU, SUR-AICU, CAR-AICU, AHDU, PICU, NICU*
- *Total surgical beds*: sum of variable *beds* with activity=S (surgical) irrespective whether adult or pediatric beds
- *Total medical beds:* sum of variable *beds* with activity=M (medicine) irrespective whether adult or pediatric beds
- *Proportion of occupied beds*: number of admitted patients in the whole hospital/ number of the beds in the whole hospital
- *Proportion of prescriptions with existing guidelines:* number of records with guideline compliance Yes or No/ number of records with guideline compliance Yes, No, NA, NI or UNK
- *Proportion of prescriptions with reason in notes*: number of records with reason in notes equal to 1 (1=Yes) / number of records with reason in notes equal to 1 (1=Yes) or 0 (0=No)
- *Proportion of prescriptions with a recorded stop/review date*: number of records with “Is a stop/review date documented” equal to 1 (1=Yes)/ number of records with “Is a stop/review date documented” equal to 1 (1=Yes) or 0 (0=No)

**At department level:**

- *Proportion of patients receiving antibiotics with parenteral antibiotic*: the proportion of patients treated at least once by administration route *P* = number of patients treated by administration route *P* / number of treated patients; **[This was excluded from the analysis]**
- *Proportion of patients receiving antibiotics with CAIs*: the proportion of treated patients with type of indication *CAI* = number of treated patients with type of indication *CAI* / number of admitted patients;
- *Proportion of patients receiving antibiotics with HAIs:* the proportion of treated patients with type of indication *HAI* = number of treated patients with type of indication *HAI* / number of admitted patients;
- *Proportion of patients receiving antibiotics with Surgical Prophylaxis*: the proportion of treated patients with type of indication *SP* = number of treated patients with type of indication SP / number of admitted patients; **[This was excluded from the analysis]**
- *Proportion of patients receiving antibiotics with Medical Prophylaxis*: the proportion of treated patients with type of indication *MP* = number of treated patients with type of indication *MP* / number of admitted patients; **[This was excluded from the analysis]**
- *Proportion of patients receiving antibiotics treated with a treatment based on a biomarker*: the proportion of patients on at least one antimicrobial whereby treatment is based on biomarker = number of patients with treatment based on biomarker equal to 1 (1=Yes) /number of patients with treatment based on biomarker equal to 1 or 0 (1=Yes, 0=No);

Remark: Temporary can be used in the analysis for the Belgian data only.

- *Proportion of patients receiving antibiotics with a targeted treatment*: the proportion of patients received at least 1 *Targeted* treatment = number of treated patients received at least 1 *Targeted* treatment / number of treated patients;
- *Proportion of admitted patients with a urinary catheter*: the proportion of admitted patients with *uc* invasive device = number of admitted patients with *uc* invasive device / total number of admitted patients;
- *Proportion of admitted patients with a peripheral vascular catheter*: the proportion of admitted patients with *pvc* invasive device = number of admitted patients with *pvc* invasive device / total number of admitted patients;
- *Proportion of admitted patients with a central vascular catheter*: the proportion of admitted patients with *cvc* invasive device = number of admitted patients with *cvc* invasive device / total number of admitted patients;
- *Proportion of admitted patients with non-invasive positive/negative mechanical ventilation*: the proportion of admitted patients with *nimv* invasive device = number of admitted patients with *nimv* invasive device / total number of admitted patients;
- *Proportion of admitted patients with an invasive respiratory endotracheal intubation*: the proportion of admitted patients with *iri* invasive device = number of admitted patients with *iri* invasive device / total number of admitted patients;
- *Proportion of admitted patients with a tubes and drains*: the proportion of admitted patients with *tubes and drains* invasive device = number of admitted patients with *tubes and drains* invasive device / total number of admitted patients;
- *Proportion of treated patients with previous non-ICU admission*: the proportion of patients on antimicrobials that were previously hospitalized, but not in ICU = number of patients on antimicrobials that were previously hospitalized (combining categories “Yes, other” and “Other”) but not in ICU / number of treated patients;
- *Proportion of patients receiving antibiotics with previous ICU admission*: the proportion of patients on antimicrobials that were previously hospitalized in ICU = number of patients on antimicrobials that were previously hospitalized in ICU (combining categories “icu” and “Yes, icu”) / number of treated patients;
- *Proportion of patients receiving antibiotics with ultimately or rapidly fatal disease (according to McCabe score)*: the proportion of treated patients with ultimately or rapidly fatal McCabe score= number of treated patients with "Rapidly fatal disease" or "Ultimately fatal disease"McCabe score / number of treated patients;
- *Proportion of patients receiving antibiotics with a surgical procedure during current admission*: the proportion of treated patients that received a surgical procedure during current admission = number of treated patients that received a surgical procedure during current admission / number of treated patients;
- *Proportion of patients receiving antibiotics with previous antibiotic use*: the proportion of treated patients with previous antibiotic use = number of treated patients with previous antibiotic use /total number of treated patients;
- *Proportion of patients receiving antibiotics of the male sex*: number of treated male patients within a department/total number of treated patients within a department
- *Proportion of patients receiving antibiotics with multiple comorbidities*: if more than 1 of the following comorbidities appears within a patient, the value of multiple comorbidities = 1:
  - Diabetes mellitus
  - AIDS/HIV
  - Hematological or solic cancer / recent chemotherapy
  - Stem cell or solid organ transplant
  - Chronic Renal Disease
  - Active tuberculosis
  - Genetic disorder
  - Congenital heart disease
  - Chronic lung diseases
  - Neutropenia
  - High dose steroids
  - Malnutrition
  - Long COVID
  - End-stage Liver Disease, cirrhosis
  - Trauma
  - Gastroenterological disease
  - Chronic neurological conditions

Further, the proportion of patients with multiple comorbidities per department will be calculated as follows: within department, number of patients with multiple comorbidities/ number of the treated patients.

## **Appendix IV –** **Estimates of the linear mixed model** to predict hospital-level prevalence of antibiotics from Global-PPS data

|  | Predictor | n | Coefficient  (95% CI) | Standard error | Degrees of freedom | T value | p value | |
| --- | --- | --- | --- | --- | --- | --- | --- | --- |
| Belgium | Proportion of antibiotic prescriptions with existing guidelines (n, %) | 1,566 (94.4%) | −3.72 (−6.72−−0.69) | 1.46 | 18.98 | −2.55 | 0.020 | |
|  | **Proportion of patients with … among patients receiving antibiotics** | | | | | | | |
|  | Previous non-ICU admission | 402 (28.6%) | 1.94 (0.66−3.21) | 0.63 | 70.00 | 3.06 | 0.003 | |
|  | **Proportion of admitted patients with … among all admitted patients** | | | | | | | |
|  | Urinary catheter | 683 (14.5%) | 2.97 (2.14−3.81) | 0.42 | 58.15 | 7.09 | 0.000 | |
| Philippines | Hospital type  (Primary or Secondary hospital) | 11 (20.0%) | 1.04  (0.42–1.67) | 0.31 | 45.37 | 3.33 | 0.002 | |
|  | Hospital type  (Infectious diseases hospital) | 2 (3.6%) | 0.72  (−0.74–2.18) | 0.73 | 51.89 | 0.98 | 0.330 | |
|  | Hospital type  (Other hospital) | 2 (3.6%) | 0.69  (−0.73–2.10) | 0.71 | 61.99 | 0.98 | 0.331 | |
|  | Proportion occupied medical beds (median, IQR) | 101 (53–180) | 2.95  (1.00–4.97) | 0.99 | 45.23 | 2.99 | 0.004 | |
|  | **Proportion of patients with … among patients receiving antibiotics** | | | | | | | |
|  | Community-acquired infections as indication | 4,949 (47.7%) | 4.14  (3.11–5.17) | 0.51 | 240.92 | 8.06 | 0.000 | |
|  | Healthcare-associated infections as indication | 1,893 (18.2%) | 3.91  (2.66–5.14)) | 0.59 | 239.41 | 6.58 | 0.000 | |
|  | **Proportion of admitted patients with … among all admitted patients** | | | | | | | |
|  | Peripheral vascular catheter | 14,302 (71.0%) | 2.25  (1.20–3.29) | 0.53 | 230.22 | 4.28 | 0.000 | |
| South Africa | **Proportion of treated patients with … among patients receiving antibiotics** | | | | | | | |
|  | Community-acquired infections as indication (n, %) | 1,865 (53.6%) | 4.70  (3.60–5.81) | 0.56 | 193.18 | 8.38 | | 0.000 |
|  | Healthcare-associated infections as indication (n, %) | 768 (22.1%) | 5.54  (4.25–6.83) | 0.66 | 190.98 | 8.45 | | 0.000 |
|  | **Proportion of admitted patients with … among all admitted patients** | | | | | | | |
|  | Urinary catheter (n, %) | 1,646 (15.1%) | 1.88  (1.16–2.61) | 0.37 | 182.90 | 5.11 | | 0.000 |
|  | Peripheral vascular catheter (n, %) | 4,592 (42.2%) | 1.97  (1.00–2.94) | 0.49 | 194.30 | 4.01 | | 0.000 |

## **Appendix V – Results of the Type III ANOVA tests** from the linear mixed model to predict hospital-level prevalence of antibiotics from Global-PPS data

|  | **Predictor** | **Sum of Squares** | **Mean Square** | **Numerator Degrees of Freedom** | **Denominator Degrees of Freedom** | **F value** | **p-value** |
| --- | --- | --- | --- | --- | --- | --- | --- |
| **Belgium** | Proportion of prescriptions with existing guidelines | 6.71 | 6.71 | 1.00 | 18.98 | 6.49 | 0.020 |
|  | **Proportion of treated patients with … among patients receiving antibiotics** | | | | | | |
|  | Previous non-ICU admission | 9.70 | 9.70 | 1.00 | 70.00 | 9.39 | 0.003 |
|  | **Proportion of admitted patients with … among all admitted patients** | | | | | | |
|  | Urinary catheter | 51.97 | 51.97 | 1.00 | 58.15 | 50.31 | 0.000 |
| **Philippines** | Hospital type | 27.23 | 9.08 | 3.00 | 52.34 | 4.09 | 0.011 |
|  | Proportion occupied medical beds | 19.85 | 19.85 | 1.00 | 45.24 | 8.95 | 0.004 |
|  | **Proportion of treated patients with … among patients receiving antibiotics** | | | | | | |
|  | Community-acquired infections as indication | 144.23 | 144.23 | 1.00 | 240.92 | 64.99 | 0.000 |
|  | Healthcare-associated infections as indication | 96.00 | 96.00 | 1.00 | 239.41 | 43.26 | 0.000 |
|  | **Proportion of admitted patients with … among all admitted patients** | | | | | | |
|  | Peripheral vascular catheter | 40.73 | 40.73 | 1.00 | 230.23 | 18.35 | 0.000 |
| **South Africa** | **Proportion of treated patients with … among patients receiving antibiotics** | | | | | | |
|  | Community-acquired infections as indication | 140.31 | 140.31 | 1.00 | 193.18 | 70.28 | 0.000 |
|  | Healthcare-associated infections as indication | 142.54 | 142.54 | 1.00 | 190.98 | 71.40 | 0.000 |
|  | **Proportion of admitted patients with … among all admitted patients** | | | | | | |
|  | Urinary catheter | 52.14 | 52.14 | 1.00 | 182.90 | 26.12 | 0.000 |
|  | Peripheral vascular catheter | 32.11 | 32.11 | 1.00 | 194.30 | 16.08 | 0.020 |

1. Versporten A, Zarb P, Caniaux I, Gros MF, et al. Antimicrobial consumption and resistance in adult hospital inpatients in 53 countries: results of an internet-based global point prevalence survey. Lancet Glob Health. 2018;**6**:e619-e629. [↑](#footnote-ref-1)
2. World Health Organization, 2015. Global Action Plan on Antimicrobial Resistance. ISBN 978 92 4 150976 3. <http://www.wpro.who.int/entity/drug_resistance/resources/global_action_plan_eng.pdf> [↑](#footnote-ref-2)
3. Pauwels I, Versporten A, Vermeulen H, et al. Assessing the impact of the Global Point Prevalence Survey of Antimicrobial Consumption and Resistance (Global-PPS) on hospital antimicrobial stewardship programmes: results of a worldwide survey. Antimicrob Resist Infect Control. 2021 Sep 28;10(1):138. [↑](#footnote-ref-3)
4. <http://www.whocc.no/atc_ddd_index/> [↑](#footnote-ref-4)
5. **Primary level:** often referred to as a district hospital or first-level referral. The hospital has few specialities, mainly internal medicine, obstetrics-gynaecology, paediatrics, and general surgery, or only general practice; limited laboratory services are available for general, but not for specialized pathological analysis. Often corresponds to general hospital without teaching function. **Secondary level:** often referred to as provincial hospital. A hospital highly differentiated by function with five to ten clinical specialities including some haematology, oncology, renal and ICU beds; takes some referrals from other (Primary) hospitals. Often corresponds to general hospital with teaching function. **Tertiary level:** often referred to as central, regional or tertiary-level hospital. A hospital with highly specialized staff and technical equipment, e.g., ICU, Haematology, Transplantation, cardio-thoracic surgery, neurosurgery and specialized imaging units; clinical services are highly differentiated by function; provides regional services and regularly takes referrals from other (primary and secondary) hospitals. Often correspond to University hospital. **Specialized hospital:** Single clinical specialty, possibly with sub-specialties; highly specialized staff and technical equipment. [↑](#footnote-ref-5)
6. Magiorakos AP, Srinivasan A, Carey RB. et al. Multidrug-resistant, extensively drug-resistant and pandrug-resistant bacteria: an international expert proposal for interim standard definitions for acquired resistance. Clin Microbiol Infect. 2012 Mar;18(3):268-81 [↑](#footnote-ref-6)
7. <https://www.who.int/features/qa/79/en/> [↑](#footnote-ref-7)
8. **A central vascular catheter** is an intravascular catheter that terminates at or close to the heart or in one of the great vessels which is used for infusion, withdrawal of blood, or hemodynamic monitoring. The following are considered great vessels: Aorta, pulmonary artery, superior and inferior vena cava, brachiocephalic veins, internal jugular veins, subclavian veins, external iliac veins, common iliac veins, common femoral veins, and in neonates, the umbilical artery/vein (ref:[http//www.cdc.gov/nhsn/pdfs/pscmanual/4psc_clabscurrent.pdf](http://www.cdc.gov/nhsn/pdfs/pscmanual/4psc_clabscurrent.pdf)) [↑](#footnote-ref-8)
9. Examples of diseases for different **McCabe score categories** (ref: <https://ecdc.europa.eu/en/healthcare-associated-infections-acute-care-hospitals/surveillance-disease-data/protocol>)

   **Non-fatal (> five years):** Diabetes; Carcinoma/haematological malignancy with > 80% five-year survival; Inflammatory disorders; Chronic GI, GU conditions; Obstetrics; Infections (including HIV, HCV, HBV – unless in above categories); all other diseases. **Ultimately fatal (One year to four years):** Chronic leukaemia, myelomas, lymphomas, metastatic carcinoma, end-stage kidney disease (without transplant); Motor neuron disease, multiple sclerosis non-responsive to treatment; Alzheimer-dementia; Diabetes requiring amputation or post amputation. **Rapidly fatal (< one year):** End-stage haematological malignancies (unsuitable for transplant, or relapsed), heart failure (EF < 25%) and end-stage liver disease (unsuitable for transplant with recalcitrant ascites, encephalopathy or varices); Multiple organ failure on intensive care unit – APACHE II score > 30, SAPS II score > 70; Pulmonary disease with cor pulmonale [↑](#footnote-ref-9)
10. Corticotherapy ≥ 30 days or recent corticotherapy at high doses (> 5 mg/kg prednisolone > 5 days) [↑](#footnote-ref-10)
11. Malnutrition refers to dietary deficiency which lead to lack of vitamins, minerals and other essential substances. Score illnesses as kwashiorkor, scurvy, delayed growth, serious underweight, etc. [↑](#footnote-ref-11)
12. Chronic neurological conditions: include Alzheimer’s disease, Parkinson’s disease, dystonia, ALS (Lou Gehrig’s disease), Huntington’s disease, neuromuscular disease, multiple sclerosis and epilepsy etc. [↑](#footnote-ref-12)
